# Damage and failure of underground subway stations under different soil constraint conditions

**DOI:** 10.1371/journal.pone.0284074

**Published:** 2023-04-06

**Authors:** Jing-Kun Zhang, Zhong-Yang Yu, Hong-Ru Zhang, Yan-Jia Qiu

**Affiliations:** 1 School of Civil Engineering, Beijing Jiaotong University, Beijing, China; 2 Key Laboratory of Urban Underground Engineering of Ministry of Education, Beijing Jiaotong University, Beijing, China; 3 The First Construction Engineering Company Ltd. of China Construction Second Engineering Bureau, Beijing, China; China University of Mining and Technology, CHINA

## Abstract

Investigations from past earthquakes have shown that underground subway stations can suffer excessive deformation under strong seismic loads, leading to the damage of critical components and the collapse of structures. This study presents the results of finite element analyses on the seismic damage of underground subway stations installed under different soil constraint conditions. The plastic hinge distribution and damage characteristics of cut and cover double-storey and three-storey subway stations are analyzed using the finite element method software ABAQUS. Combined with the static analysis results of the column sections, a discriminant method for bending plastic hinges is presented. The numerical results show that the collapse of the subway stations begins with the failure of the bottom columns’ bottom sections, which leads to the bending of the plates and the destruction of the whole structure. The bending deformation at the end section of columns has an approximatively linear relationship with the inter-storey drift ratio, and the change in soil conditions shows no apparent influence. The sidewall deformation behavior varies significantly under different soil conditions, and the bending deformation at the bottom section of sidewalls increases along with an increase in the soil-structure stiffness ratio at the same inter-storey drift deformation level. The sidewall bending ductility ratio of the double-storey and three-storey stations at the elastic-plastic drift ratio limit increases by 61.6% and 26.7%, respectively. In addition, the fitting curves between the component bending ductility ratio and inter-storey drift ratio based on the analysis results are also presented. The findings may provide a helpful reference for the seismic performance evaluation and design of underground subway stations.

## 1 Introduction

**Fig 1:**
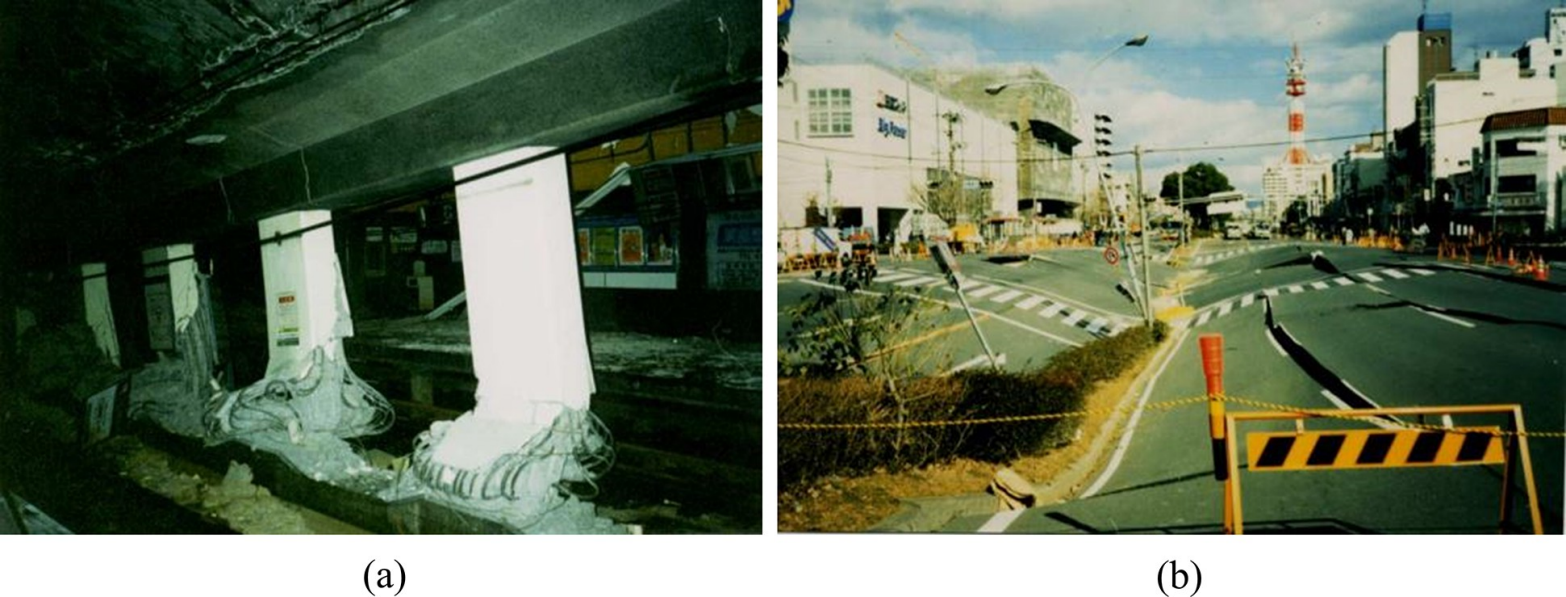
Damage to the Daikai station in the Kobe earthquake [2]. (a) Failure of center columns and collapsed roof, (b) Substantial surface settlement caused by the collapse of the Daikai station underneath.

Underground rail transit has become an essential part of China’s urban public transport network. However, almost all the cities that have opened or are planning to open subway lines in China are located in seismic fortification zones, so the seismic resistance of underground stations is significant. The severe damage to the Daikai Station, as presented in Fig 1, during the 1995 Kobe earthquake and many other subsequent seismic damage cases of underground structures have caused engineering circles to pay increasing attention to the seismic design of underground structures [1–4]. Moreover, the post-earthquake repair of underground structures is more complicated and costly than that of aboveground structures, so higher requirements are put forward for the study of seismic damage to underground structures.

Furthermore, the dynamic response characteristics of underground structures are quite different from those of aboveground structures, which is mainly associated with the fact that underground structures are constrained by the surrounding soil. When an earthquake occurs, underground structures deform along with the surrounding soil, while the vibration characteristics of the structures are not among the main influence factors in their seismic responses. In contrast, historical seismic damage records and related studies confirm that soil deformation plays a significant role in controlling the seismic behavior of underground structures, and it can be tremendously affected by site soil conditions [5–8]. Therefore, numerous studies have been conducted to explore soil conditions’ influence on seismic responses and damage to underground structures. Most of these studies focus on the structure’s acceleration and inter-storey drift deformation response. Only a few pieces are concentrated on the damage and failure characteristics of multi-storey and multi-span underground structures under different soil constraint conditions. In this regard, Zhuang et al. [9, 10] and Yang et al. [11] analyzed the response law and damage characteristics of multi-storey subway station structures under different site types through the finite element method (FEM). Li et al. [12] analyzed the difference in deformation and acceleration distribution between the Daikai Station and the Nagata Station during the Kobe earthquake, the influence of soil conditions on the damage and failure of structures and the causes of the different damage degrees of the two stations were discussed. Wang et al. [13] studied the seismic response of an unequal-span subway station under different site conditions and presented the distribution of structural tensile damage and its variation rule with site conditions. Rezaiee-Pajand et al. [14–18] suggested several new types of finite element and analytical methods that can be used for dynamic and damage analysis of underground structures. Shirkhani et al. [19–23] studied the seismic damping systems for structures and evaluated their effectiveness. In addition, the dynamic soil characteristics and seismic response law of underground structures under special soil conditions were also investigated through various research methods by some scholars, such as liquefiable soil [24–28], soft soil [29–33], and other special soils [34–36].

Referring to the seismic damage records of the Daikai station and other underground structures, the key to preventing complete collapse is to ensure that the vertical load-bearing components can maintain sufficient vertical bearing capacity under the horizontal structural deformation induced by earthquakes. Unlike aboveground structures, the seismic load borne by underground structures mainly comes from the deformation of the surrounding soil, making the transmission path of seismic load and the characteristics of internal force in components more complex and changeable than those of aboveground structures. This complicates the relationship between the damage degree of vertical load-bearing components and the horizontal deformation of underground structures. To date, existing research on the damage of underground structures under different soil conditions is mainly concentrated on the structure level, such as the deformation response of the structure or the distribution of the damage. The damage characteristics of the critical components that may cause a complete collapse are not paid enough attention to, especially for the deformation and performance requirements of the vulnerable parts of the vertical load-bearing components under different soil conditions and seismic loads.

In addition, since some of the current design methods for underground structures are derived from that for aboveground structures, the inter-storey drift ratio is also employed as an evaluation index of seismic performance for underground structures in many studies, which was initially for aboveground structures. Nevertheless, underground and aboveground structures’ deformation modes and stress distributions are quite different under earthquakes. Whether a unified seismic performance evaluation of underground structures can be given by the index of inter-storey drift ratio under different soil conditions remains to be further explored.

In this study, typical cut and cover double-storey and three-storey underground subway station structures are taken for research, and a total of 300 analysis cases are constructed by designing different soil conditions and ground motion inputs. A nonlinear numerical analysis model considering the soil-underground structure interaction is established using the FEM, combined with implicit and explicit subroutines of reinforced concrete materials. The occurrence and development characteristics of bending plastic hinges are investigated based on the static analysis results of critical vertical load-bearing structural component sections. In addition, fitting functions are presented for predicting the bending deformation at the end section of columns and sidewalls of subway station structures with different soil-structure stiffness ratios. The underground station damage process and failure mechanism under different soil constraint conditions are also discussed through the element deletion method.

The remainder of this paper is organized as follows. Firstly, the basic information and the establishing method of the numerical analysis model are introduced in detail. Then, the discriminant method of plastic hinges for fiber beam elements is covered in detail. Subsequently, the computational results are presented and analyzed, including the development of plastic hinges and the bending deformation of critical components. Finally, conclusions and suggestions for future research are presented.

## 2 Numerical analysis model

This section introduces the numerical model’s establishing method and analysis procedure in detail, including the material constitutive models and their validation, the initial stress balancing method, the boundary conditions, and the relevant analysis parameters.

### 2.1 Material constitutive model and failure criterion

#### 2.1.1 Constitutive model for soil

**Fig 2:**
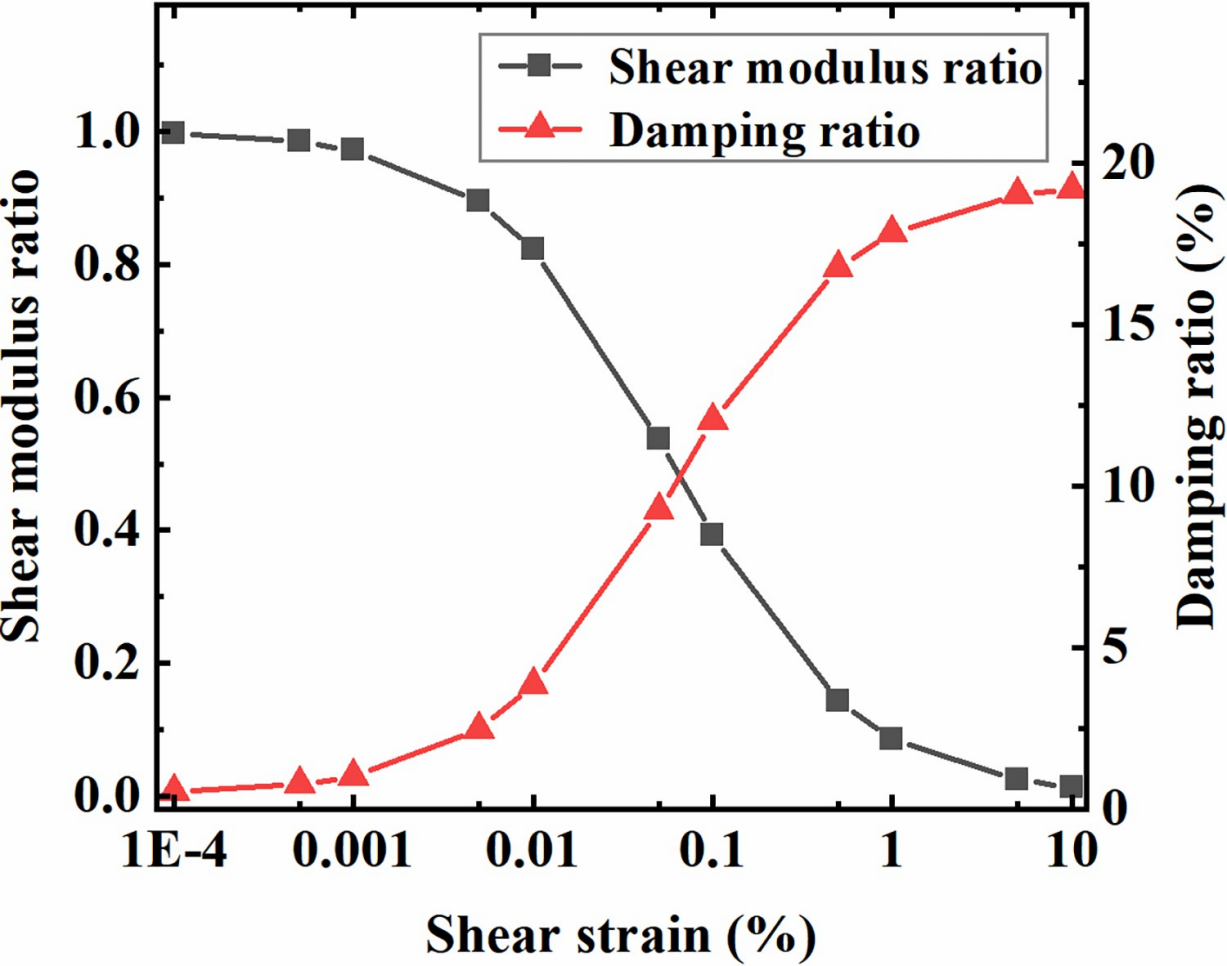
Dynamic shear modulus ratio and damping ratio with the dynamic shear strain of the soil.

In the proposed study, the Drucker-Prager model is adopted for the constitutive model of soil. The soil properties are determined by the equivalent linear method based on frequency domain analysis to approximately characterize the nonlinear properties of soil under dynamic loads. Specifically, the equivalent shear modulus and damping ratio of soil are calculated according to the variation curves of soil shear modulus and damping ratio with its shear strain. Iterative calculations are carried out by constantly updating the soil material parameters until they meet specific accuracy requirements. During this step, the one-dimensional equivalent linear site response analysis program EERA is employed. The soil of various sites adopts the same shear modulus ratio and damping ratio curves, as shown in Fig 2 [37].

The equivalent damping of soil is represented by the Rayleigh damping as follows:

C=αM+βK (1)

where ***C*** is the damping matrix, ***M*** is the mass matrix, ***K*** is the stiffness matrix, and *α* and *β* are the mass and stiffness damping coefficients, respectively.

The Rayleigh damping is associated with frequency, which can be reflected by the coefficients *α* and *β*. For the damping ratio of any vibration mode, it can be expressed by:

$$\zeta_{n}=\frac{\alpha }{2\omega_{n}}+\frac{\beta \omega_{n}}{2}$$ (2)

where *ζ*_n_ and *ω*_n_ denote the damping ratio and natural frequency under a certain vibration mode, respectively.

Through the equivalent linear method, the equivalent damping ratio *ζ*_eq_ of soil can be extracted, which can be brought into Eq (2) to obtain the following equation:

$$\left\{\begin{array}{c} \alpha \\ \beta \end{array}\}=\frac{2\zeta_{\mathrm{eq}}}{\omega_{i}+\omega_{j}}[\begin{array}{c} \omega_{i}\omega_{j}\\ 1 \end{array}\right]$$ (3)

The damping coefficients *α* and *β* can be determined by selecting the appropriate natural frequency *ω*_i_ and *ω*_j_. Considering that the seismic response of soil-underground structure system is dominated by low-order vibration mode, the first-order natural frequency of site and the dominant frequency of input motion are adopted as *ω*_i_ and *ω*_j_ respectively, as suggested by Du et al. [38] in their study. It is worth mentioning that the equivalent soil shear modulus obtained from the equivalent linear analysis is adopted as the shear modulus of the site soil when calculating its first-order natural frequency.

#### 2.1.2 Constitutive models for structure

**Fig 3:**
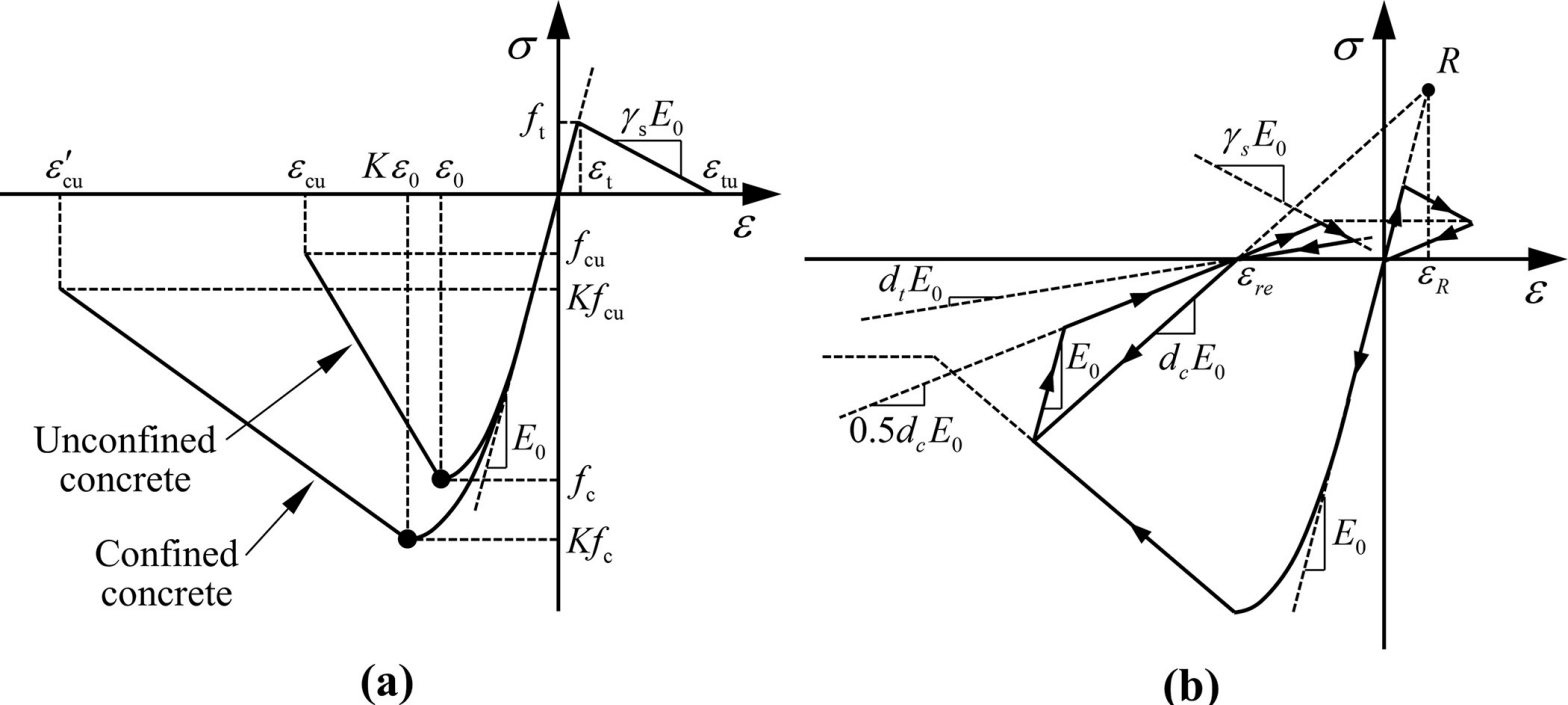
Uniaxial constitutive model for concrete. (a) Stress-strain envelope curve, (b) Loading-unloading criteria.

The constitutive models for concrete and reinforcement are realized by Fortran subroutines. The modified Kent-Park uniaxial constitutive model [39] is adopted for concrete, whose envelope curve and loading-unloading criteria are illustrated in Fig 3. In the figure, *E*_0_ is the initial stiffness under compression, *f*_c_ is the axial compressive strength, *f*_cu_ is the residual compressive strength, and *f*_cu_ = 0.2 *f*_c_. *f*_t_ is the axial tensile strength, and *γ*_s_ is the softening coefficient of tensile stiffness, which is 0.1. *ε*_0_ and *ε*_t_ are the strains corresponding to the peak compressive and tensile strength, respectively. *ε*_cu_ and *ε*^′^_cu_ are the ultimate compressive strains of unconfined and confined concrete, respectively. *K* is the improvement coefficient of confined concrete, and *d*_c_ and *d*_t_ are the compressive and tensile damaged stiffness coefficients, respectively.

**Fig 4:**
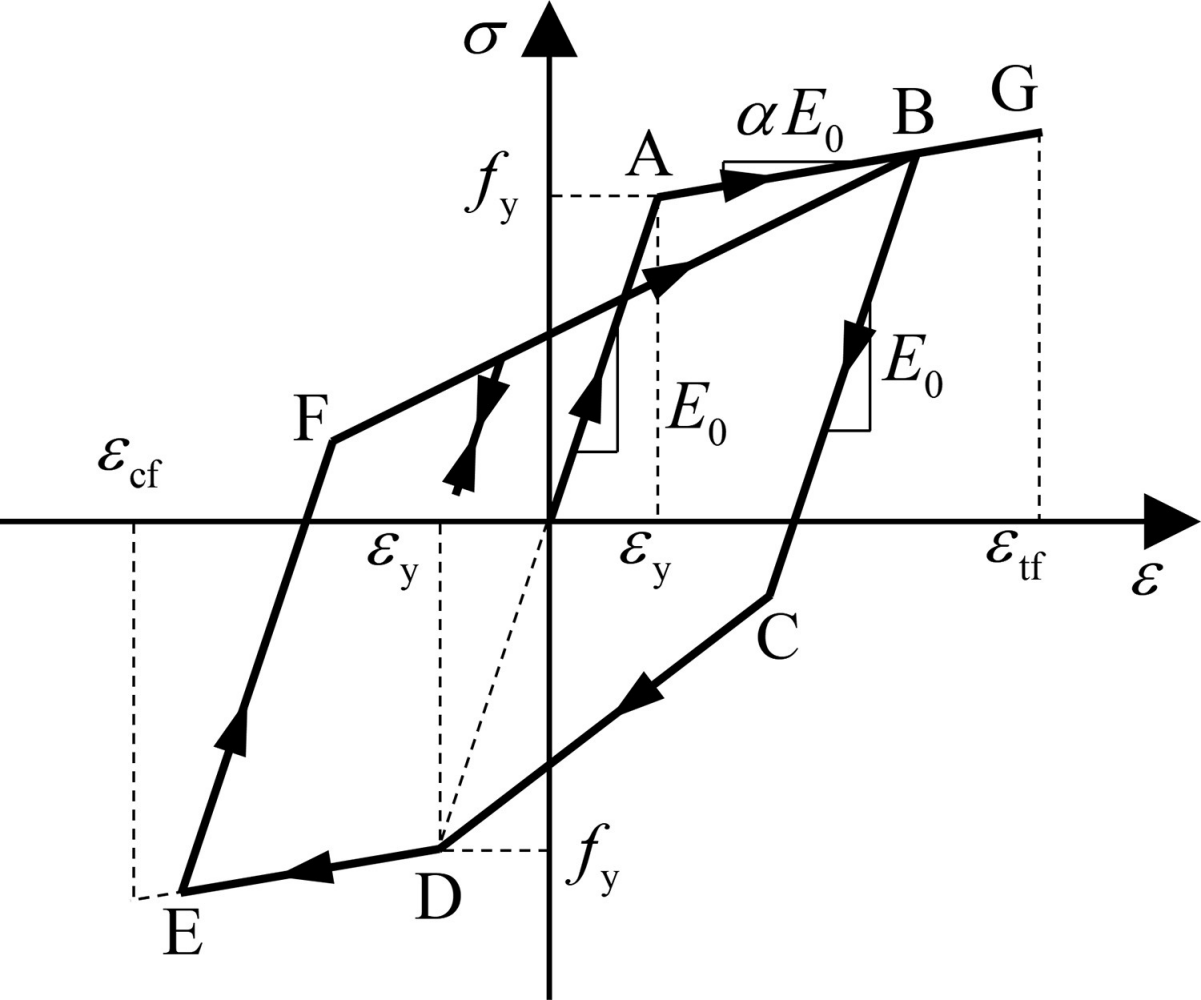
Uniaxial constitutive model for reinforcement.

The constitutive model for reinforcement adopts a bilinear model with loading-unloading criteria of the Clough model [40], as shown in Fig 4, and the tensile and compressive failure of the reinforcement is also considered. In the figure, *α* is the postyield stiffness coefficient, which is 0.001, and *E*_0_ is the initial stiffness.

#### 2.1.3 Material failure criterion

Under the action of strong earthquakes, the stress and strain of the concrete and reinforcement at critical sections of underground structures will increase sharply. Afterward, concrete crushes and reinforcement fractures or buckling failure occurs, leading to the structural components’ transformation into independent parts and the loss of their bearing capacity. To replicate the above damage characteristics, this study adopts the element deletion method to control the failure of sections during analysis by setting corresponding failure criteria in concrete and reinforcement material subroutines. For concrete material, it is considered that when its compressive strain reaches the ultimate compressive strain *ε*_cu_ (*ε*^′^_cu_ for confined concrete), as illustrated in Fig 3, compressive failure occurs, and concrete material failure is identified at this point. When a structural component is subjected to bending moment, the tensile load of the cross-section is generally borne by the longitudinal reinforcement due to the low tensile capacity and ultimate tensile strain of the concrete material, and the tensile capacity of the section is considered lost when the reinforcement is subjected to tensile fracture. Therefore, the tensile failure strain *ε*_tu_ of the concrete material adopts the tensile failure strain of the reinforcement, that is, *ε*_tf_.

For the reinforcement, fracture failure is assumed when the tensile strain reaches the ultimate tensile strain *ε*_tf_, as illustrated in Fig 4, which is set to 50 times the yield strain *ε*_y_ [41]. When the concrete around the reinforcement reaches the ultimate compressive strain *ε*_cu_, it is considered that it lost the ability to wrap the reinforcement, which leads to the reinforcement failure due to compressive buckling, and hence the ultimate compressive strain *ε*_cf_ of the reinforcement is assumed as equal to *ε*_cu_.

#### 2.1.4 Validation of constitutive models

**Fig 5:**
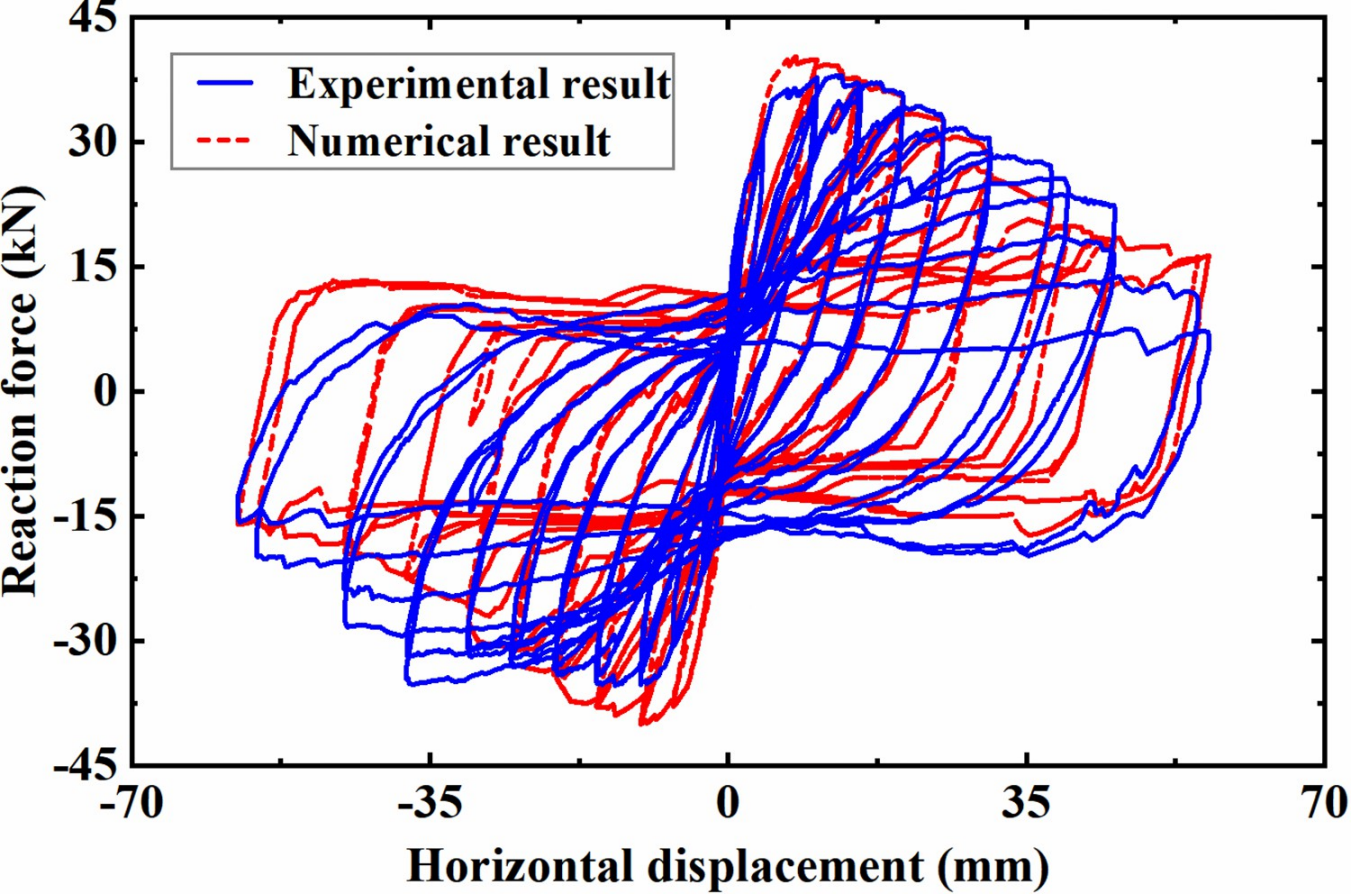
Comparison between the experimental result and numerical analysis result.

To validate the above constitutive models for concrete and reinforcement, a numerical analysis model of a cantilever column in the literature [42] is established, and the corresponding quasi-static pushover test is reproduced and analyzed. Fig 5 shows the comparison between the experimentally measured horizontal reaction force of the actuator and that extracted from the numerical simulation at the corresponding position. As seen from the figure, when the loading amplitude is relatively low, the reaction force extracted from the numerical simulation is in good agreement with the experimentally measured value, which can successfully reproduce the loading and unloading process of the component. With the loading amplitude increased, the strength and stiffness results of the numerical simulation are slightly different from the measured results. It is extrapolated to be associated with the angle existing between the vertical actuator and the column axis at high loading amplitude, which leads to the biased measurement of the column axial compression ratio. Moreover, the damage of the concrete base connecting with the column is not considered in the analysis, which further increases the divergence. Generally, the material constitutive models adopted in this study can realistically replicate the mechanical properties and damage process of reinforced concrete structural components under a complex loading and unloading environment.

### 2.2 Input ground motion

**Fig 6:**
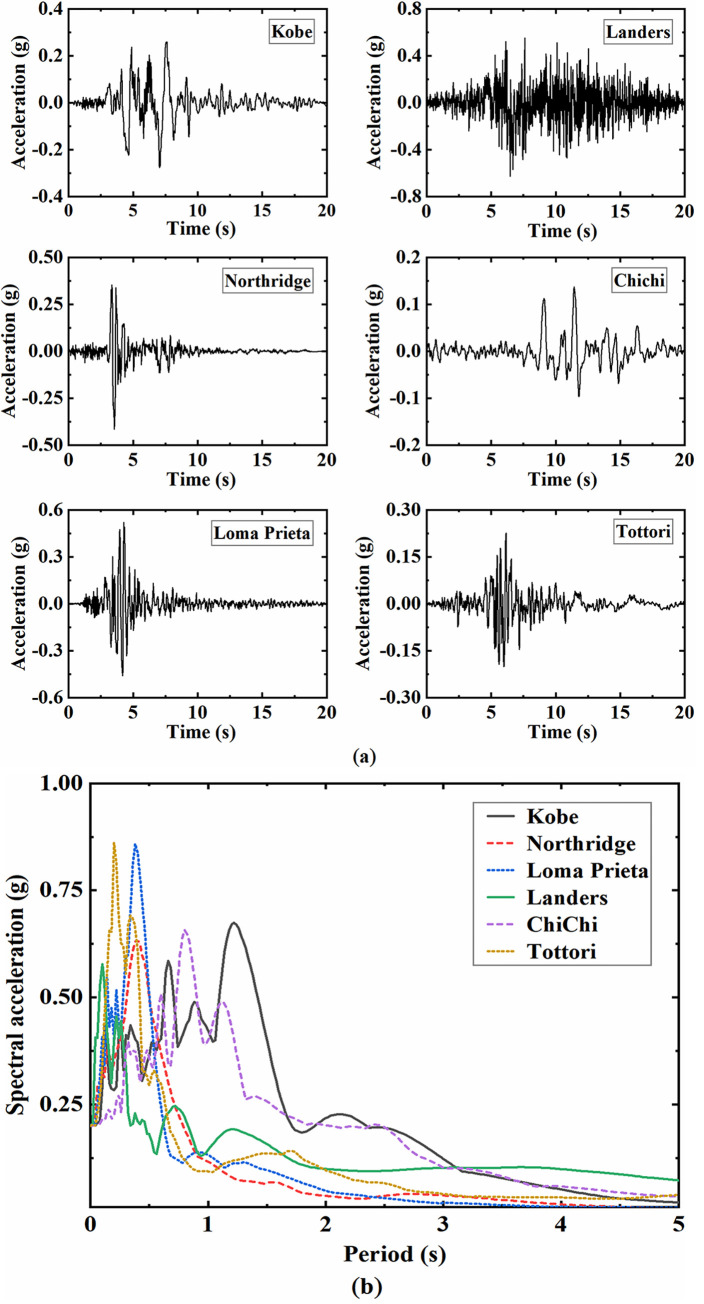
Time-history curves and response spectra of input seismic waves. (a) Time-history curves of acceleration, (b) Acceleration response spectra (with 5% damping and peak acceleration of 0.2g).

The spectral characteristics of seismic waves are essential factors affecting the seismic response of the site soil and underground structures. In this study, seismic waves are selected from the Pacific Earthquake Engineering Research Center (PEER) ground motion database as the input ground motions. The basic information of the selected earthquake records and the components adopted in the analysis are summarized in Table 1, whose acceleration time-history curves and response spectra are presented in Fig 6. The 30 m equivalent shear wave velocities of the sites where the seismic records were acquired are all above 950 m/s, and hence they can be approximately regarded as bedrock ground motion records, which are imposed on the bottom boundary of the numerical model after filtering. In addition, the peak accelerations of selected seismic waves are scaled between 0.2 g to 1.2 g to investigate the dynamic response and damage characteristics of underground structures under different intensities of seismic motions.

**Table 1:** Information of ground motion records.

Earthquake	Year	Location	Component	*V*_30_ (m•s^-1^)	Type
Kobe	1995	Hyogo, Japan	KBU000	1043.0	Near-field
Northridge	1994	California, USA	PAC175	2016.1	Near-field
Loma Prieta	1989	California, USA	G01090	1428.1	Near-field
Landers	1992	California, USA	LCN260	1369.0	Near-field
Chichi	1999	Taiwan, China	HWA003N	1525.9	Far-field
Tottori	2000	Tottori, Japan	SMNH10EW	967.3	Far-field

### 2.3 Dynamic analysis procedure and boundary conditions

**Fig 7:**
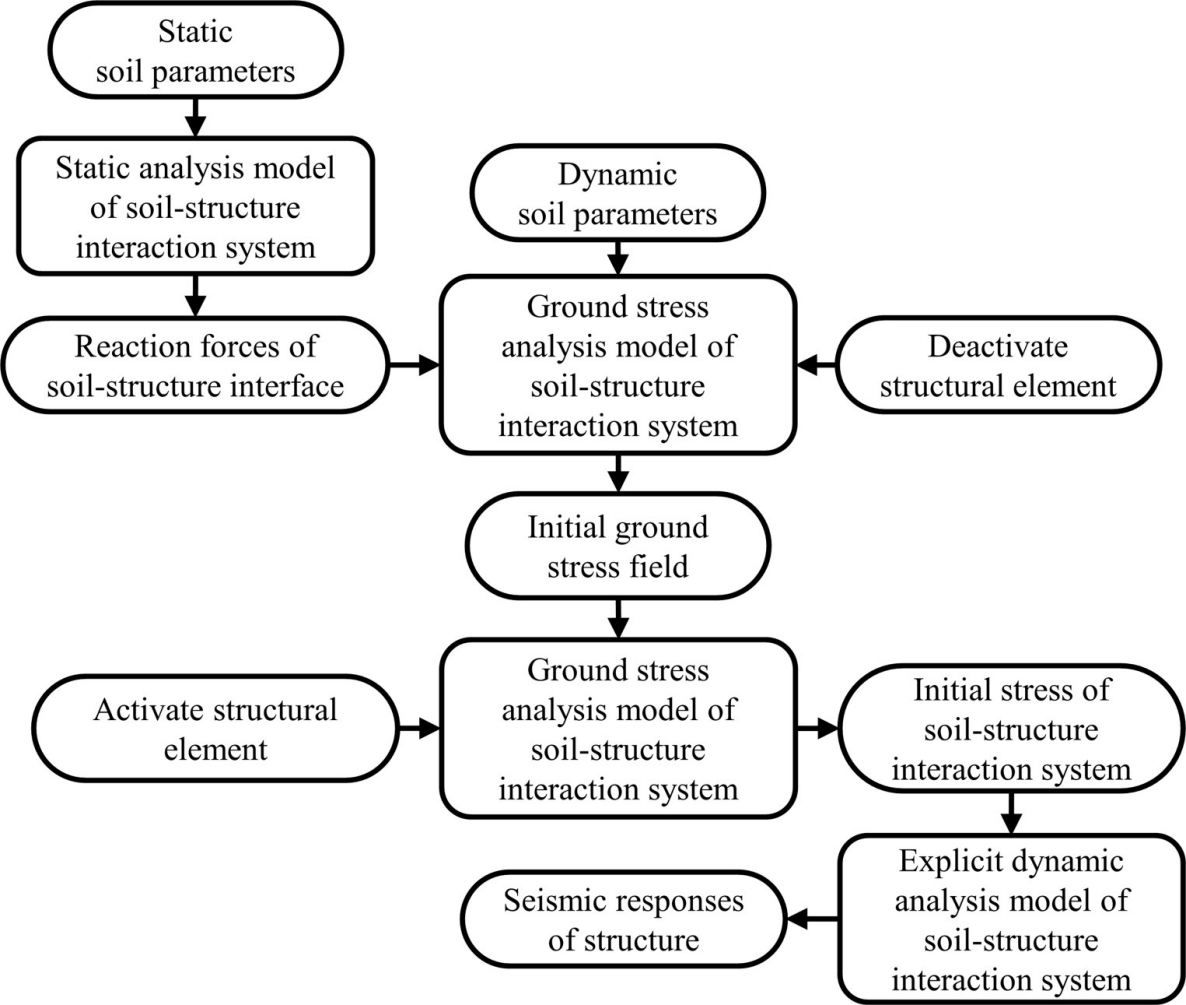
Procedure of the dynamic analysis method.

**Fig 8:**
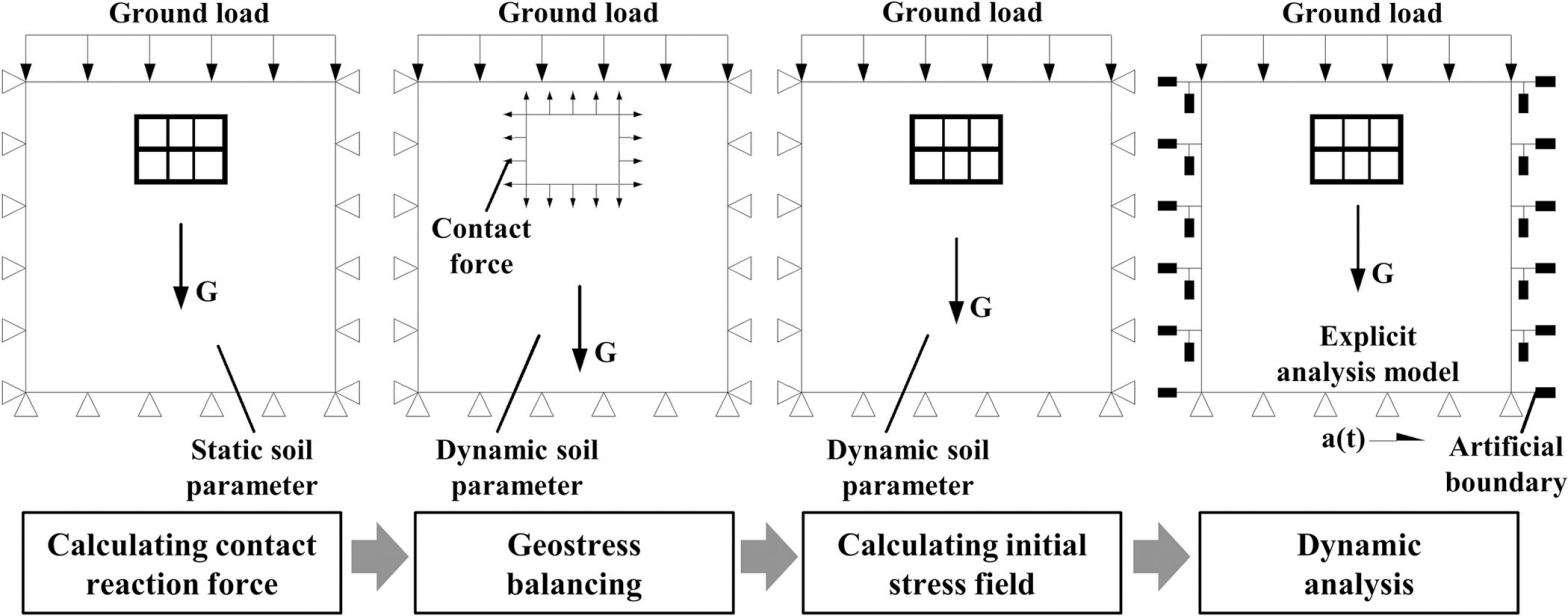
Loads and boundary conditions during the analysis.

The initial ground stress in the soil-underground structure system is an essential prerequisite for underground structure seismic response analysis. When the equivalent linear method is employed to characterize the nonlinear properties of soil, the equivalent dynamic soil parameters are generally adopted to solve the initial stress field, which will lead to the diverseness in the initial stress states of soil and structure under different seismic loading conditions. Therefore, unified static soil mechanical parameters are adopted in this study to solve the initial stress of the site soil, and the results are implemented in the subsequent explicit dynamic analysis. The specific analysis procedure is explained in Fig 7, and the loads and boundary conditions of the numerical model in each step of the analysis are illustrated in Fig 8.

It is worth mentioning that the strain of the beam element at the final increment step in the static analysis is stored as a solution dependent state variable (SDV) and transferred to the explicit dynamic analysis module to ensure the continuity of structural element strain in the transfer process. Moreover, consistent constitutive models and variable definitions are adopted in the UMAT and VUMAT subroutines for precisely transmitting beam element stress and strain between the implicit solver and explicit solver.

In order to minimize the reflection of input waves at the boundaries of the computational domain, as shown in Fig 8, energy transmitting and viscous boundaries are adopted along the lateral boundaries of the explicit dynamic analysis model by introducing spring and dashpot elements to replicate the infinite environment of site soil, as suggested by Lysmer and Kuhlemeyer [43]. The free field response is also considered as force boundary conditions along the lateral boundaries. Since the bottom of the numerical model is assumed to be the surface of the underlying bedrock, seismic waves are input at the bottom boundary in the form of horizontal acceleration, where no energy transmission boundaries are adopted.

### 2.4 Establishment of the numerical model

**Fig 9:**
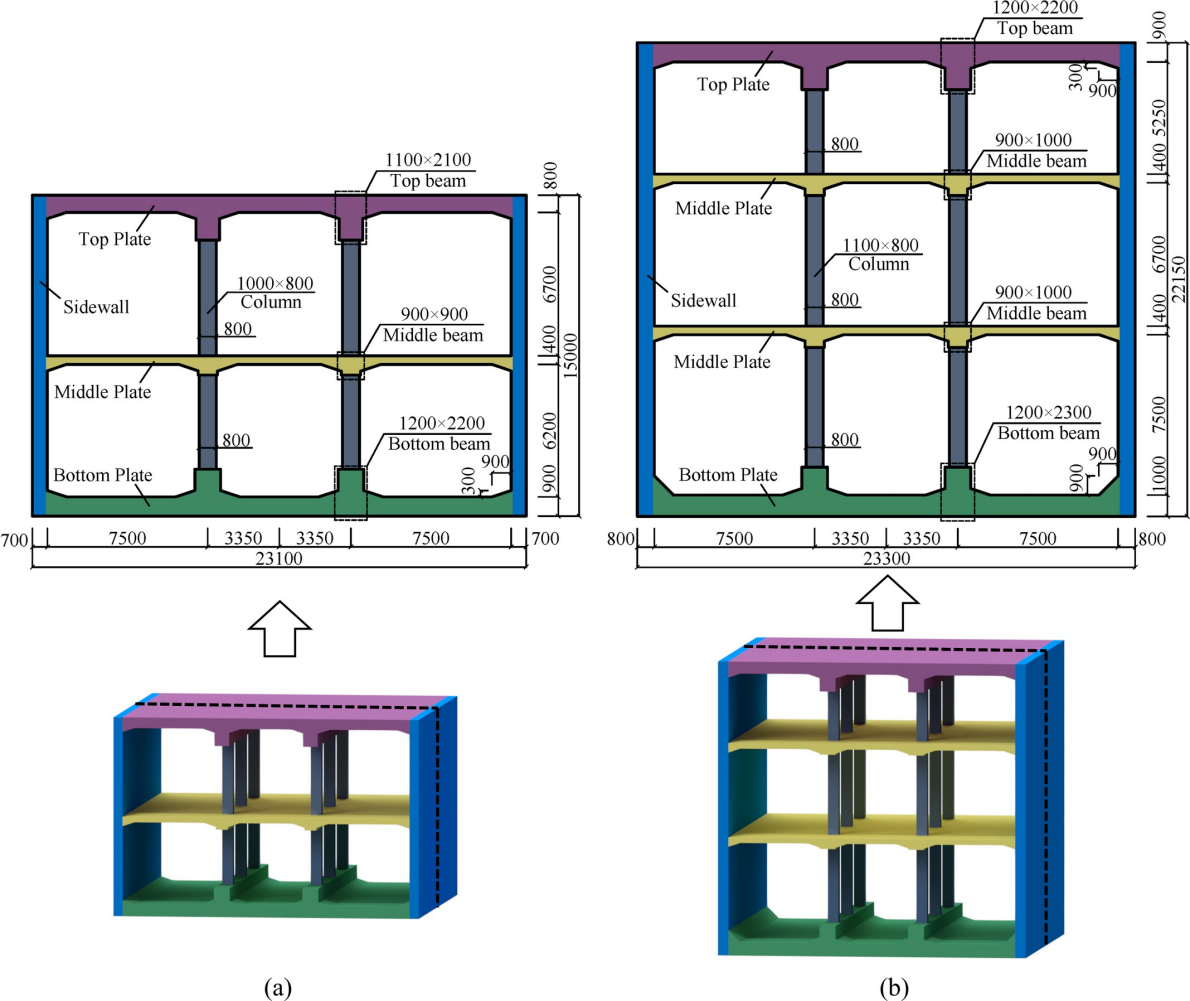
Cross-section of the underground structures (mm). (a) Double-storey station, (b) Three-storey station.

In this study, typical cut and cover double-storey and three-storey underground subway station structures with double columns and three spans are numerically modeled for analysis, and their standard sections are simplified as a plane strain problem. The total heights of the two stations are 15.0 m and 22.15 m, and the total widths are 23.1 m and 23.3 m. The section size of each component is illustrated in Fig 9, in which the columns space at every 9.12 m. The stations are provided with longitudinal beams at the joints between columns and plates, and the joints between beams, sidewalls, and plates are arranged with haunches.

The station structures are made of C45 concrete. HRB400 and HPB300 grade steels are used for the longitudinal reinforcement and the stirrup of the columns, respectively. The material parameters are determined according to the Code for Design of Concrete Structures (GB 50010–2010). The station structures adopt symmetrical reinforcement, and the reinforcement ratio of each main component is summarized in Table 2, in which the volume stirrup ratio of the columns is 0.75%. The thickness of the covering soil layer above the top plate is 4.0 m, and the surface overload is 20 kPa. The decoration, equipment, and crowd loads on the middle and bottom plates are 16 kPa in total. Under static load, the maximum axial compression ratios of the double-storey and three-storey station columns are 0.58 and 0.61, respectively.

**Table 2:** Reinforcement ratio of the underground structures.

Component	Ratio (%)	Component	Ratio (%)
Top plate	1.2	Sidewall	1.4
Middle plate	1.0	Column	1.6
Bottom plate	1.1		

**Fig 10:**
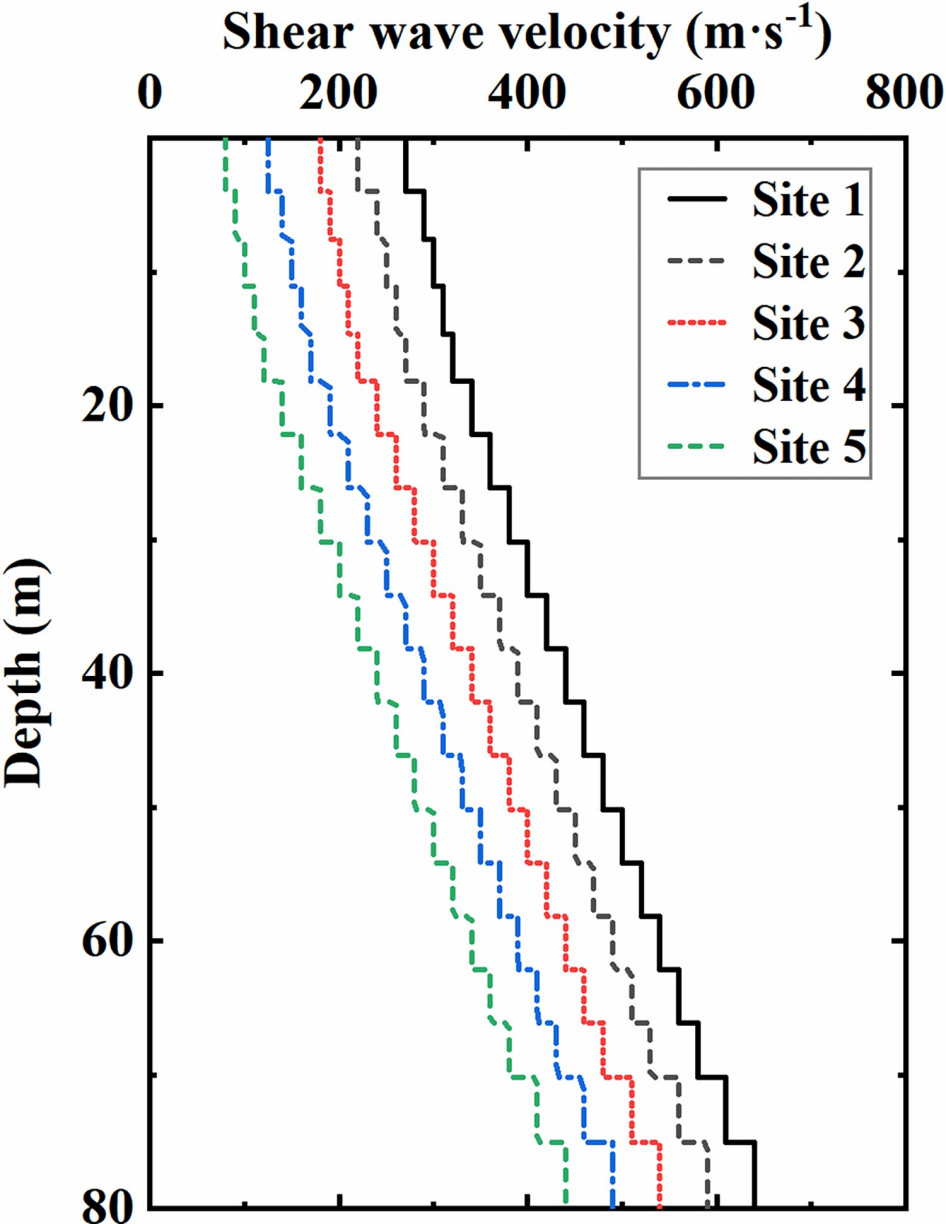
Variation in shear wave velocity with the soil depth.

The sites in the analysis are composed of sandy soil, whose thickness is 80 m, and the underlying layer is assumed to be rigid bedrock. Regardless of the influence of soil stratification and earthquake liquefaction, the main soil parameters are summarized in Table 3. Moreover, five different soil conditions are constructed in this study by adjusting the soil shear wave velocity, as plotted in Fig 10. The soil-structure stiffness ratio is adopted to quantify the constraint ability of surrounding soil on underground structures, as shown in Table 4, which is calculated according to the definition given by Wang [44]:

$$F=\frac{GL}{HS_{1}}$$ (4)

where *G* is the horizontal shear deformation stiffness of soil at the corresponding position of the underground structure, *L* is the width of the structure, *H* is the height of the structure, and *S*_1_ is the horizontal force required to produce unit relative displacement between the top and the bottom of the structure.

**Table 3:** Soil parameters.

Parameter	Value	Parameter	Value
Density (kg•m^-3^)	2000.0	Dilation angle (°)	15.0
Cohesion (kPa)	3.0	Poisson’s ratio	0.26
Friction angle (°)	29.0		

**Table 4:** Soil-structure stiffness ratio *F* of different site soil conditions.

Site type	Double-storey station	Three-storey station
Site 1	10.26	9.48
Site 2	7.19	6.80
Site 3	4.66	4.56
Site 4	2.68	2.75
Site 5	1.23	1.37

The detailed two-dimensional numerical analysis model is developed in the general FEM software ABAQUS. The bottom boundary is set to the bedrock surface, meaning the model’s total height is 80 m. The model’s total width is taken as 250 m, which is approximately 11 times the width of the station, to ensure that the lateral boundaries have a minimal influence on the underground structure’s dynamic response. The out-of-plane thickness of the model is set as the longitudinal spacing of the columns, that is, 9.12 m. The interfaces between the structure and surrounding soil are defined as frictional contacts with a friction coefficient of 0.4 in the tangential direction, and separation is allowed in the normal direction.

**Fig 11:**
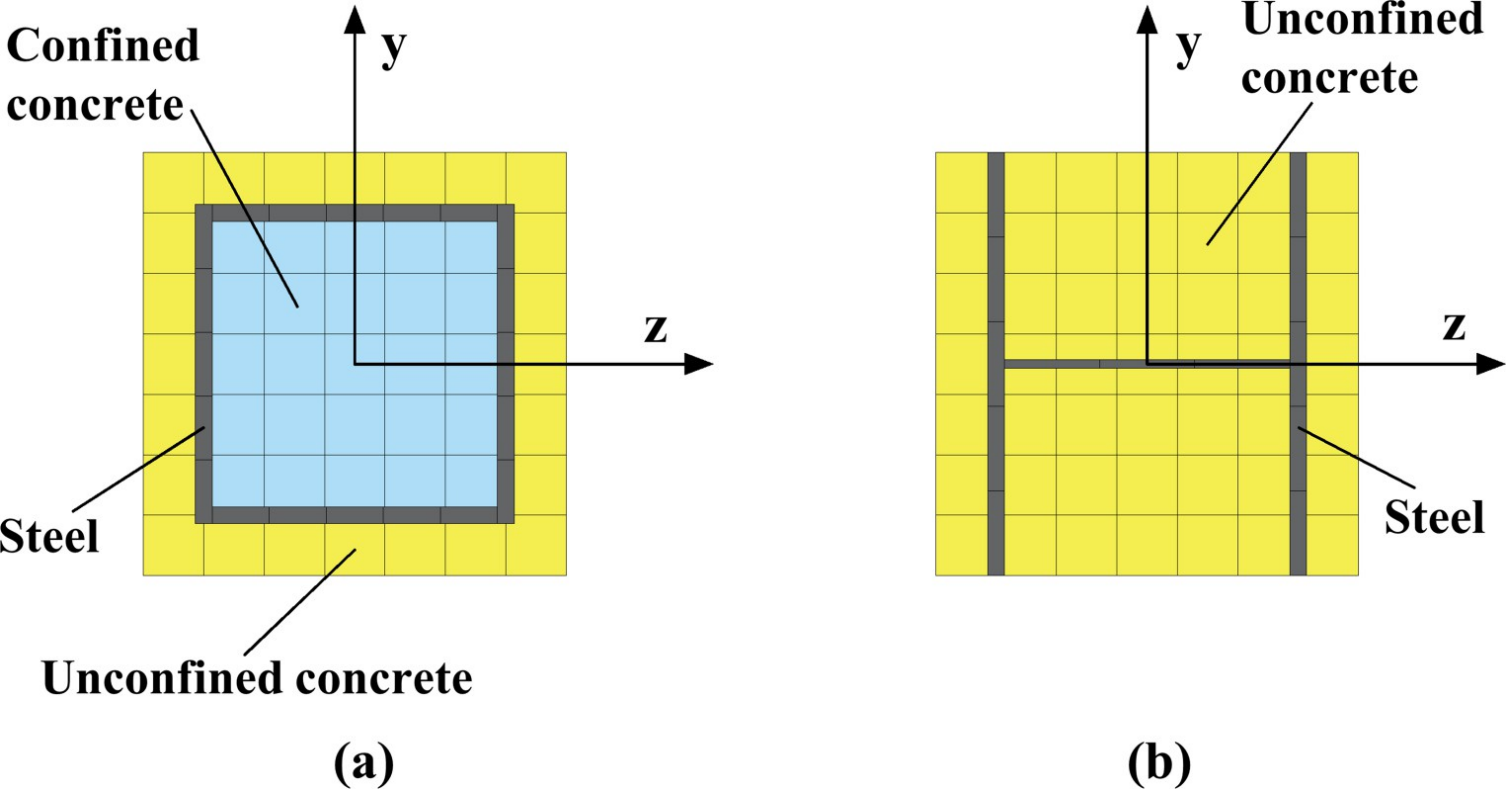
Diagram of fiber distribution. (a) Column section, (b) Sidewall section.

The soil is meshed with 4-node reduced integral plane strain elements (CPE4R). The mesh size is less than 1/8 of the wavelength of the seismic waves’ maximum frequency to ensure that all frequency bands can be reproduced effectively, and the soil elements near underground structures are refined. The Timoshenko fiber beam elements (B21) are adopted for the structural components, and the dimension of the beam elements is the same as the height of the corresponding component cross-section. The components’ intersecting areas are assumed rigid, and the top, middle, and bottom longitudinal beams are considered elastic, whose damage is not covered in this study. The reinforcement is modeled by establishing shared-node stringer elements, meaning that the concrete and reinforcement elements coordinate their deformation. The slip between reinforcement and concrete is not considered. The section shape of the reinforcement element is determined by equivalent conversion according to the distribution and cross-sectional area of the reinforcement. The web thickness of the equivalent I-shaped sidewall reinforcement element section adopts a tiny value so that its effect can be ignored, and the concrete section is divided into 19×19 fibers, as depicted in Fig 11.

## 3 Discrimination of the bending plastic hinge on vertical load-bearing structural components

Under seismic loading, the formation and development of bending plastic hinges can effectively reflect structures’ damage degree and failure process. However, for vertical load-bearing structural components, the joint action of axial and bending loads makes it possible to form different failure patterns, and hence it is difficult to judge the section yield simply by the working state of longitudinal reinforcement. In experimental studies, the yield of structures or structural components can generally be determined on the load-deformation curves by specific discriminant methods [45–47]. Similarly, this study adopts static load analysis to obtain the bearing capacity of component sections under different working conditions and forms a method to identify the bending yield of vertical load-bearing components and further calculate the corresponding damage index.

### 3.1 Section analysis method

Prior to conducting the dynamic analysis, the load-deformation characteristics of the column and sidewall under different axial compression ratios are statically analyzed separately with the concrete and reinforcement constitutive models elaborated above. The fiber beam element adopted in this study is not coupled with axial force and bending moment in the calculation of shear force and shear deformation, and the discrimination of bending plastic hinges is generally at the component section level. Hence, the influence of component length is not considered here. The column and sidewall numerical models are developed with fixed constraints at the bottom, axial force and rotational displacement applied on the top. Afterwards, the rotational displacement load is gradually increased while the axial load is kept constant to extract the bending moment and corresponding curvature of the bottom section of the structural component, based on which the load-deformation relationship of the component section under a specific axial compression ratio can be described. The moment-curvature curves under different axial compression ratios are obtained by changing the axial load on the top. It is worth mentioning that the shear damage and failure of the component section are not covered in the analysis, considering that a component with a rational shear design can improve its shear performance to a large extent and avoid premature shear failure.

### 3.2 Bending bearing characteristics and yield discrimination of column section

#### 3.2.1 Bending bearing characteristics of column section

**Fig 12:**
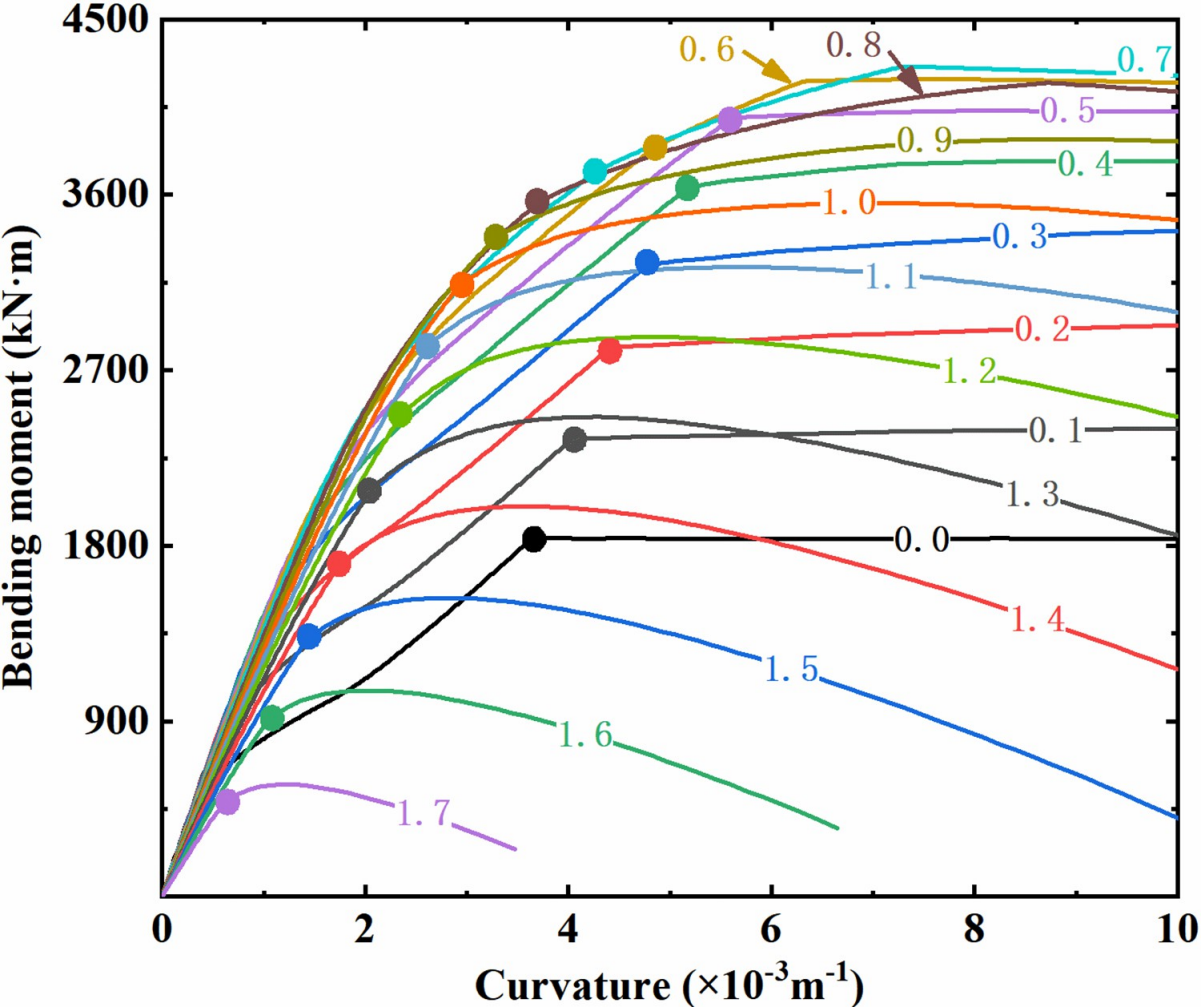
Bending moment-curvature curves of the column section with different axial compression ratios.

Taking the column of the double-storey station as an example, the moment-curvature relationship of the column section under various axial compression ratios is depicted in Fig 12, where the axial compression ratios are labeled on the curves. It can be observed from the figure that the bending bearing capacity shows a trend of first increasing and then decreasing with increasing axial compression ratio, indicating that a moderate axial load can improve the bending bearing capacity of components to some extent. In addition, when the axial compression ratio is relatively low, the sectional bending moment increases with increasing curvature at first and remains approximately constant after an obvious turning point. At this point, the longitudinal reinforcement yields while the concrete in the compressive region of the column section is slightly damaged. That is, it presents a form of tensile failure. However, when the axial compression ratio is relatively high, the bending moment-curvature curves have no obvious turning points, and rapid drops are observed in the section bending moment after reaching the peak. The concrete in the compressive region of the column section is seriously damaged, leading to a significant compressive capacity reduction. Meanwhile, the reinforcement is in a state of tension or compression but does not yield. That is, it presents a failure form of compression. For the two sectional failure forms with obviously different mechanical performances, the "farthest point method" is adopted in this study to achieve unified discrimination on their bending yield based on the macroscopic mechanical behavior of the component section [46]. The point on the curve farthest from the line between the original point and the peak point is regarded as the yield point, and its coordinates can be expressed as follows:

$$(\theta_{y},M_{y})=\underset{\left(\theta_{y},M_{y})=(\theta ,M\right)}{\max\;d}$$ (5)

$$d=\frac{\left|M_{p}\cdot \theta -M\cdot \theta_{p}\right|}{\sqrt{M_{p}^{2}+\theta_{p}^{2}}}$$ (6)

where (*θ*, *M*) is the coordinate of any point on the bending moment-curvature curve of the component section, (*θ*_y_, *M*_y_) is the yield point coordinate, (*θ*_p_, *M*_p_) is the coordinate of the peak point of the curve, and 0≤*θ*≤*θ*_p_.

#### 3.2.2 Section yield discrimination of column section

**Fig 13:**
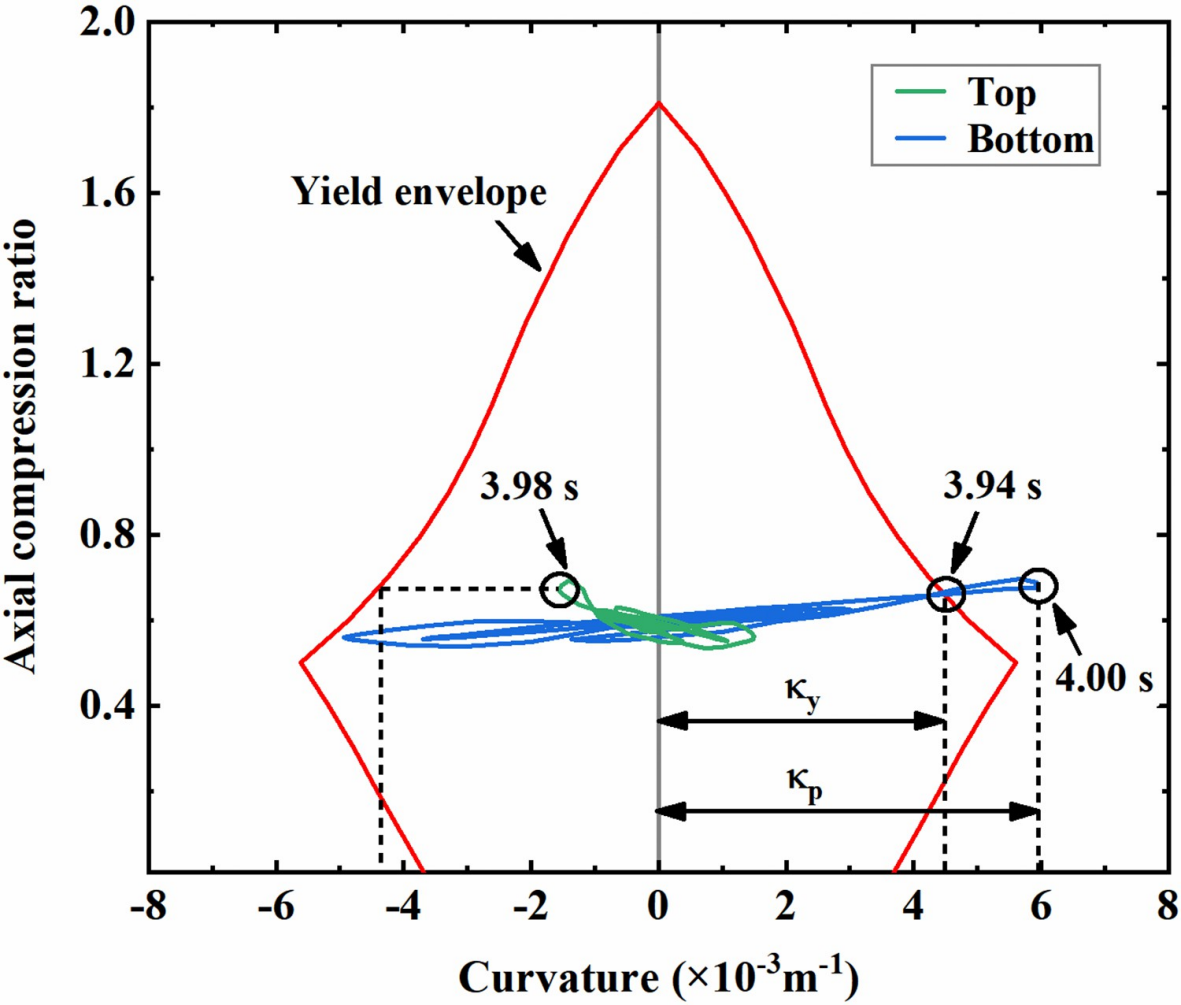
Yield curvature envelope of the column section.

By the method above, the column section’s yield points at different axial compression ratio levels are marked with solid dots in Fig 12. As shown, the yield bending moment and the corresponding curvature of the column section both increase first and then decrease with an increase in the axial compression ratio. Based on the yield section curvature under different axial compression ratios, the envelope curve for discriminating the component section yield can be obtained, as illustrated in Fig 13. A plastic hinge is identified to form at the corresponding section when its curvature response extracted from the dynamic analysis exceeds the yield curvature envelope.

Fig 13 visualizes the curvature responses of the double-storey station’s bottom column sections under the 0.3g Northridge wave, which are selected for a detailed explanation of the method. As shown, the curvature amplitude of the column’s bottom section is significantly larger than the top, indicating that the bottom section is more susceptible to seismic damage. For the bottom section, the curvature response reaches the yield envelope at 3.94 s after the beginning of the earthquake, which denotes the section yield and the formation of a bending plastic hinge. At this moment, the section curvature reaches 0.00449 in specific, which can be noted as the yield curvature of the section, namely, the *κ*_y_ identified in Fig 13. As bending deformation progressively increases after the section yields, the curvature response reaches the peak at 4.00 s, which is 0.00593 in specific, as indicated by the *κ*_p_ in Fig 13. However, for the top section of the column, the curvature response remains within the yield envelope during the entire earthquake process. The curvature response reaches its peak of 0.00156 at 3.98 s, which is far below the corresponding yield curvature of 0.00442 under the axial compression ratio at this point. That is, the top section does not yield under the seismic load. Based on this method, the bending plastic hinges of columns are identified, and the damage degree of sections is quantitatively analyzed later in this study.

### 3.3 Bending bearing characteristics and yield discrimination of sidewall section

**Fig 14:**
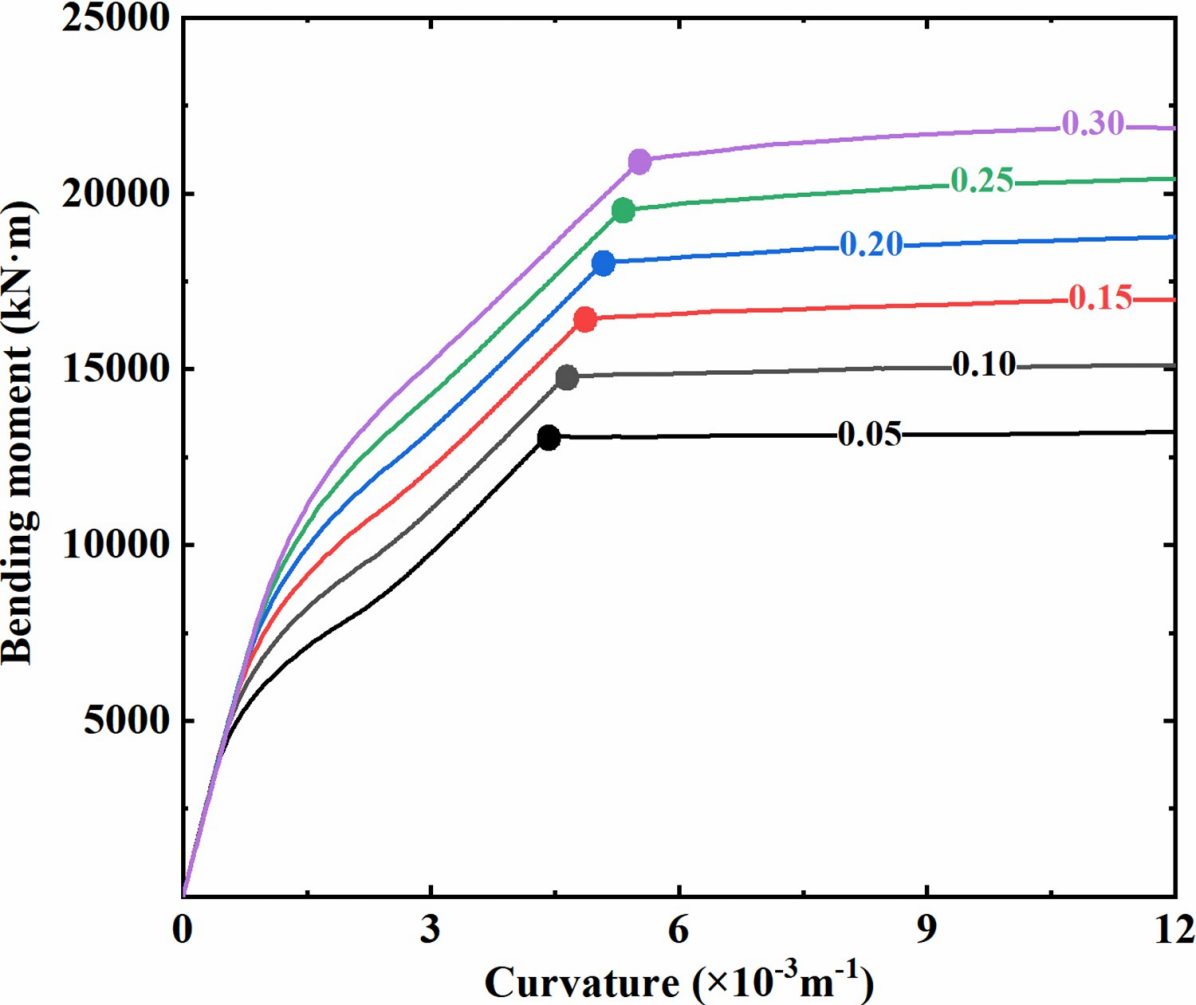
Bending moment-curvature curves of the sidewall section with different axial compression ratios.

**Fig 15:**
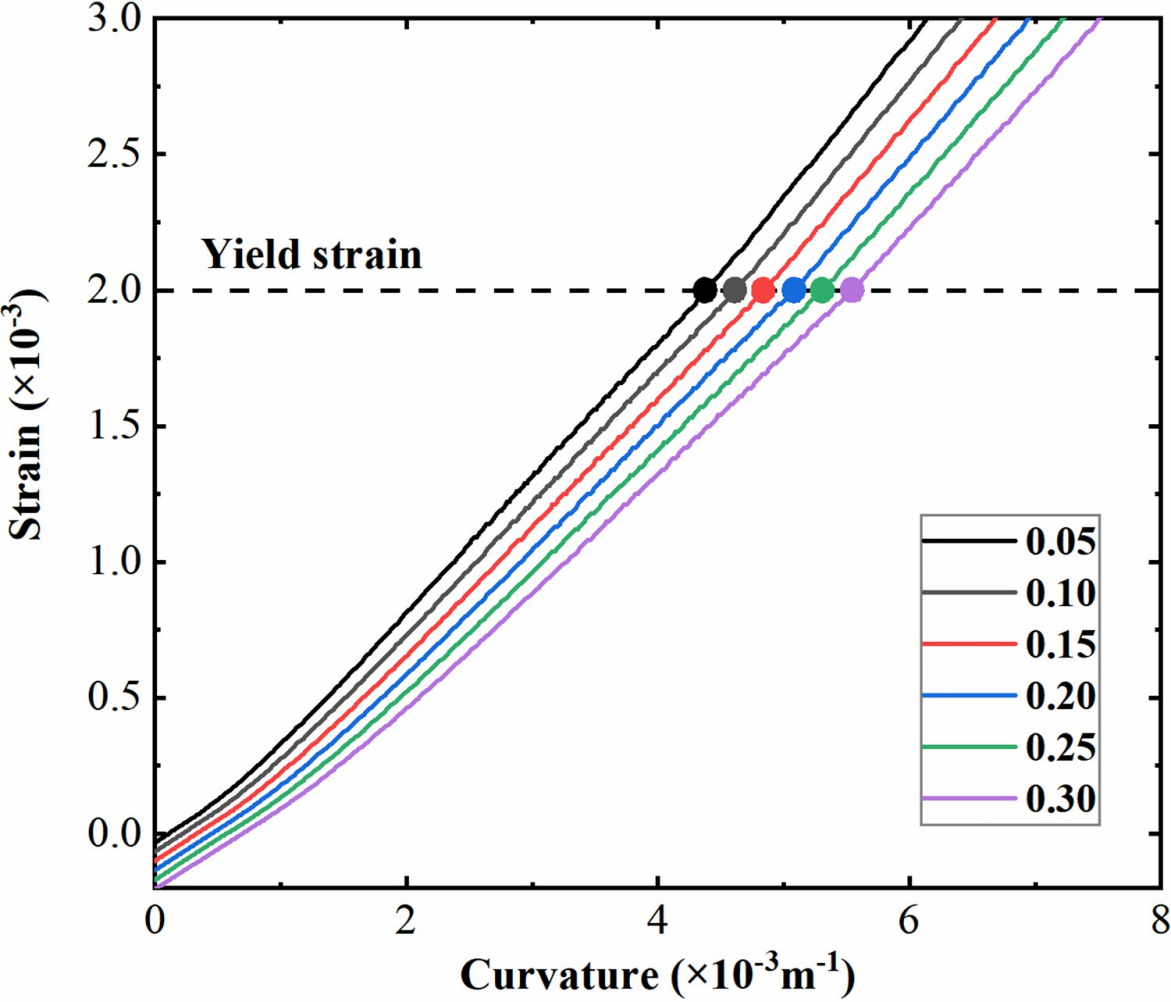
Strain of tensile reinforcement of the sidewall section with different axial compression ratios.

Similarly, with the double-storey station as an example, the moment-curvature relationship of the sidewall section with various axial compression ratios is plotted in Fig 14, where the axial compression ratios are labeled on the curves. As the cross-sectional area of the sidewall is much higher than that of the column, its axial compression ratio generally stays at a relatively low level under normal circumstances, and hence only the cases with an axial compression ratio below 0.3 are considered here. In the same way, the yield points of the sidewall section under each axial compression ratio are marked with solid dots in Fig 14. Similar to the column section with a relatively low axial compression ratio, the yield bending moment of the sidewall section shows a gradual increase with an increase in the axial compression ratio, reflecting the improvement of the axial load on the bending capacity of the component section. In addition, each bending moment-curvature curve has an obvious turning point, which is consistent with the tensile failure characteristics described above. As illustrated in Fig 15, the strain of the tensile reinforcement of the sidewall section under each axial compression ratio is further extracted, and the particular strains at the section yield points are marked with solid dots according to Fig 14. It can be seen that when the sidewall section yields, the strain of the tensile reinforcement reaches the yield level simultaneously. The sidewall section still presents the form of tensile failure due to its relatively low compression ratio, even though it is subjected to the combined action of axial load and bending moment. Therefore, the reinforcement yield can be adopted as the criterion for discriminating the bending plastic hinge for the sidewall section.

## 4 Influence of soil constraint conditions on the damage of underground structures

This section explores the influence of soil constraint conditions on the damage of various components by comparing the formation process and development degree of bending plastic hinges on subway station structures and the deformation characteristics of critical component sections between different analysis conditions.

### 4.1 Structural damage process and failure pattern

Under strong seismic motions, the component sections of underground structures will experience substantial plastic deformation. With increasing deformation, the sectional bearing capacity grows slowly or remains nearly constant, and at this point, a bending plastic hinge is formed. Based on the distribution and development of plastic hinges with incremental input ground motion intensity, this section presents the structures’ weak positions and damage process. The failure pattern of the underground stations is also discussed.

#### 4.1.1 Structural damage process

**Fig 16:**
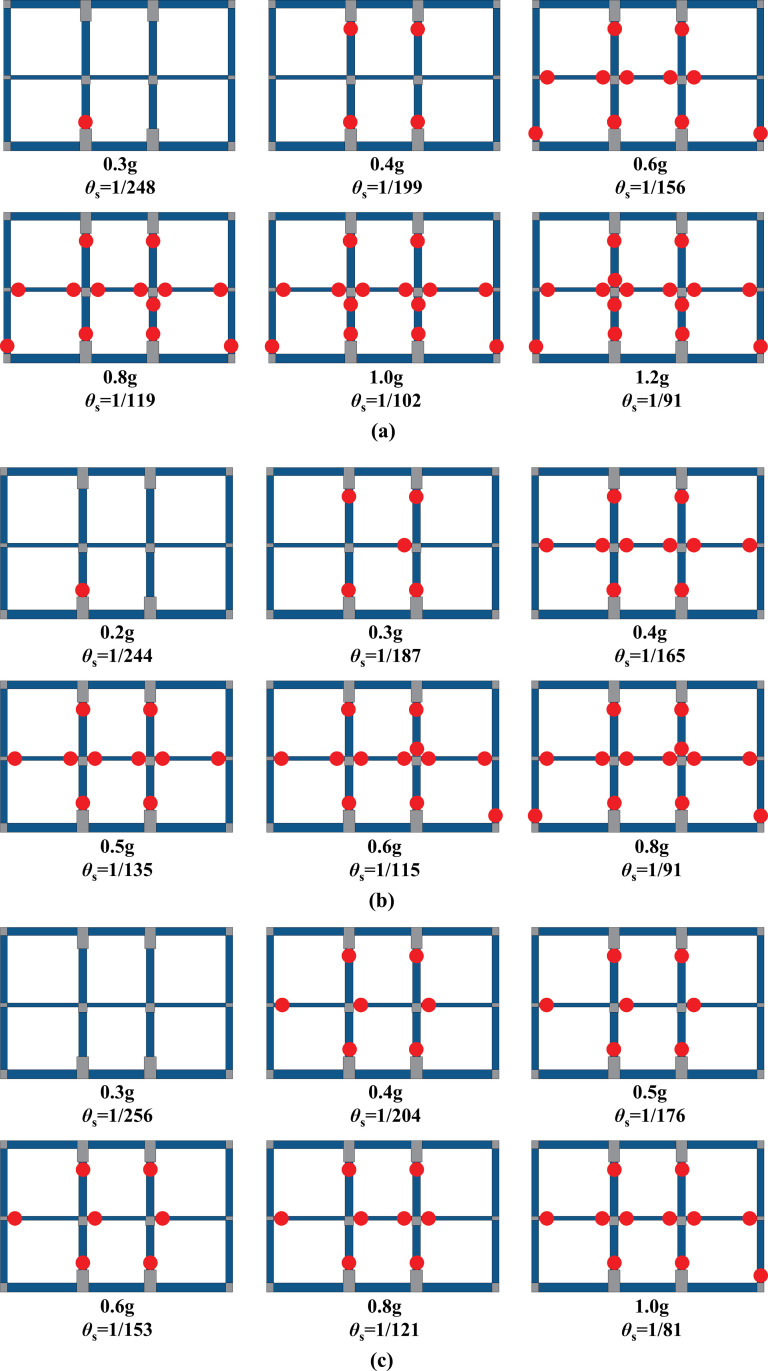
Plastic hinge distribution under the Northridge wave. (a) Site 1, *F* = 10.26, (b) Site 3, *F* = 4.66, (c) Site 5, *F* = 1.23.

With the double-storey station structure as an example, the distributions of bending plastic hinges after the action of the Northridge wave with different intensities at each site and the corresponding peak roof drift ratio *θ*_s_ (calculated as the relative lateral displacement of the structure divided by the total height of the structure) are shown in Fig 16. The solid red dots represent plastic hinges discriminated by the method described above. The blue area represents the reinforced concrete component meshed with fiber beam elements, and the gray area represents the component joints and longitudinal beams whose damage is not considered. In addition, the soil-structure stiffness ratio of each site is presented for the convenience of comparison, and only partial results with similar roof drift ratios are selected due to space limitations. As illustrated in Fig 16, when the peak acceleration of input motion reaches 0.2 g, the value of *θ*_s_ is approximately 1/250. Although slight plastic deformation is observed in some regions of the structure, most component sections are not yield, except the bottom column’s bottom section at site 1 and site 3. With increasing input motion intensity, plastic hinges gradually appear in the joint areas between columns and beams, and the number of plastic hinges at the middle plate continues to increase. Further raising the input intensity until *θ*_s_ reaches approximately 1/90, the plastic hinges are widely distributed on the structure. However, the vertical load-bearing components still maintain sufficient bearing capacity at this moment, and the internal space of the station structure remains nearly intact.

#### 4.1.2 Structural damage characteristics

Based on the development process of the bending plastic hinges of structural components under various analysis conditions, some common damage and failure characteristics can be summarized:

1. When the structure is damaged, the surrounding soil plays a prominent role in constraint and support. Under strong seismic motions, the soil around the underground station structure not only imposes the seismic load on the structure but also limits the deformation of the structure to a certain extent. Once the structure is seriously damaged, the support of the surrounding soil prevents it from collapsing sideways. As shown in Fig 16, most components yield at the end sections when *θ*_s_ reaches 1/90 except for the top and bottom plates. At this time, widely distributed plastic hinges significantly reduce the lateral deformation stiffness of the station structure and make it approximately geometrically deformable. Nevertheless, the structure still maintains a relatively complete internal space under the support and constraint of the surrounding soil, and there is no sign of complete collapse. Similar phenomena were also observed by Jing et al. [48] and Chen et al. [49] in their experimental studies on the seismic damage of multi-storey subway stations. This indicates that it is insufficient to adopt lateral deformation stiffness as the criterion for the complete failure of underground structures.
2. The columns are the critical factor affecting underground station structures’ aseismic performance. Fig 16 shows that the bending plastic hinge first appears at the end of the columns. Because the cross-sectional area of columns is normally limited in design by the requirement of using space, underground structures’ column axial compression ratio is generally much higher than that of aboveground structures. The high axial compression can easily induce a sudden drop in the bearing capacity due to sectional damage, leading to the structure’s chain failure reaction, as confirmed by Nam et al. [50], Ma et al. [51] and Dong et al. [52] in their studies on the seismic damage of the Dakai station in the Kobe earthquake. Moreover, plastic hinges at the column end appear after that at the middle plate end, as illustrated in Fig 16. This damage pattern is more akin to the beam hinge failure mode, although it should be classified as mixed hinge failure mode by definition (i.e., beam and column hinges exist simultaneously). It is in accord with the design purpose of "a strong column with a weak beam" for aboveground frame structures, which is beneficial for the aseismic performance. In contrast, for column-top plate joints and column-bottom plate joints, the cross-sectional area and reinforcement amount of the plates are much larger than those of the columns. Hence, they all present a typical column hinge failure mode. It is also worth mentioning that the analogous buckling column failure, as in the case of the Daikai Station, is not observed during the analysis. It is speculated to be associated with the low column slenderness ratios in this study (6.8 and 5.5 for the top and bottom columns of the double-storey station, respectively; 4.9, 7.6 and 7.0 from the top to the bottom columns of the three-storey station, respectively). The bearing capacity of the columns is mainly controlled by the sectional bearing capacity rather than the compression stability. Therefore, section failure occurs at the column ends, which experience the most significant seismic response.
3. The soil condition has an apparent influence on the damage characteristics of underground station structures. As mentioned above, the surrounding soil simultaneously plays the role of seismic load transferring and lateral supporting. Its mechanical properties directly affect the dynamic response of underground structures and play a prominent role in the damage characteristics of structures. With the sidewall as an example, Fig 16 reveals that the damage degree of the bottom section decreases with decreasing soil-structure stiffness ratio. Specifically, bending plastic hinges can be found at the bottom sections of both sidewalls when the roof drift ratio reaches 1/156 at site 1 (*F* = 10.26), while the bottom sections of the sidewalls still remain not yielded even the roof drift ratio reaches 1/121 at site 5 (*F* = 1.23). Taking the column-middle plate joint in Fig 16 as another example, it can be found that the joint area presents an appropriate beam hinge failure mode at site 5. The column sections remain not to yield even the roof drift ratio reaches 1/81, which means that the middle plate fully participates in the seismic energy dissipation with plastic deformation and delays the damage to the columns. In contrast, plastic hinges appear at the columns soon after the middle plate yields at site 1, which leads to the premature decline of the column bearing capacity and reduces the global aseismic performance of the structure. This indicates that different soil conditions can significantly change underground structures’ deformation mode and internal force distribution so that the structures present different damage states even if the inter-storey drift ratios are at the same level, which is compatible with the conclusions given by Li et al. [12] and Wang et al. [13].

#### 4.1.3 Structural failure pattern

**Fig 17:**
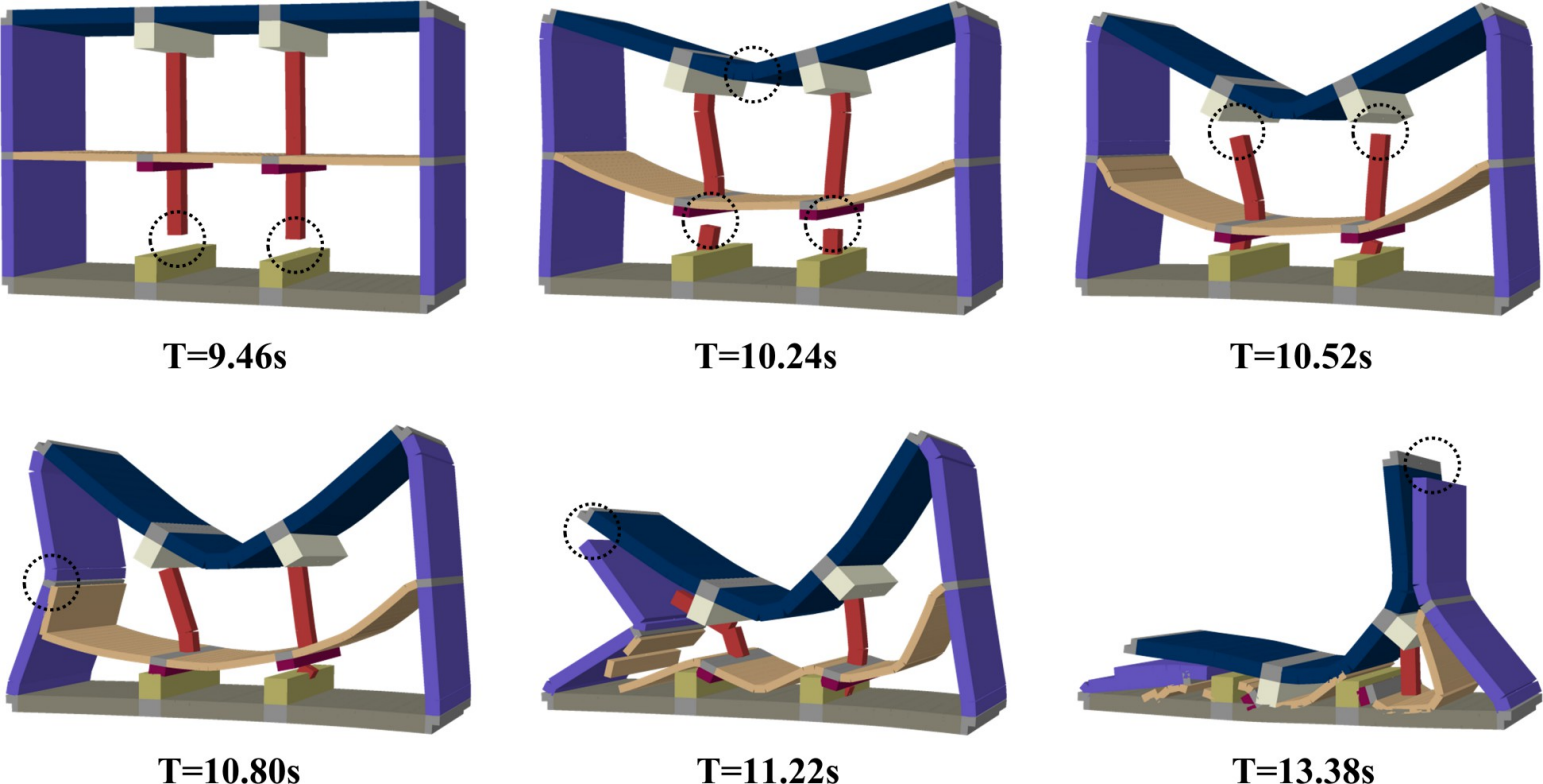
Collapse process of the subway station structure.

When the intensity of the input seismic wave is further increased, the whole station structure collapses. Here, the double-storey station at site 4 under the Northridge wave is taken as an example to explain its structural failure pattern. Before the destruction, the roof drift ratio of the structure reaches 1/46. The collapse process is illustrated in Fig 17, and the soil elements are hidden to better demonstrate the structural deformation. As shown, the whole collapse process starts at 9.46 s after the earthquake begins, with the bottom column’s bottom sections first failing under the combined action of axial load and lateral deformation. Subsequently, the plates that lost vertical support begin to suffer downward settlement under self-weight and overburden loads, and apparent bending deformation is observed in the middle span of the top plate. Then, the fractured bottom columns hit the bottom plate, while the severely damaged top sections ultimately fail, which causes both bottom columns to be separated from the main structure at 10.24 s. With the top plate continuously sinking, the top section of the top columns also fails under massive deformation at 10.52 s. The top plate completely loses vertical support, which aggravates its bending deformation. Furthermore, bending failures are observed likewise at the joint areas between sidewalls and middle plates at 10.80 s. Afterwards, the joint areas between sidewalls and top plates are damaged at 11.22 s as the bending angle of sidewalls continuously increases, after which the top, middle, and bottom plates of the left half of the structure entirely overlap due to the loss of vertical support. The right half leaves only a small amount of space under the support of the remaining part of the sidewall and top plate. At 13.38 s, the structure completely collapses, as shown in Fig 17.

The damage process and failure pattern of the station structure have some similarities with the well-known case of the Daikai station. For example, both stations’ collapse starts with column failures, leading to the bending of upper plates and roofs. However, significant differences also exist between the two cases. For the station structure in the proposed study, severe bending damages are observed in the middle of the sidewalls, which were not reported in the seismic damage investigations of the Daikai station [2]. This is speculated to be associated with the fact that the sidewall height in this study is relatively higher, resulting in a more significant seismic load and bending deformation on the sidewalls compared to that of the Daikai station. At the same time, the plates that lost vertical support settle downward and bend after the failure of the bottom columns, resulting in the loss of original horizontal support to the sidewalls. Moreover, the bended plates induce inward loads on the sidewalls, which further aggravates the bending damage. This explains the reason that the sidewall bending damage occurs near the position where they are connected with the plate components. The damage process under other analysis conditions is similar and will not be described and discussed here.

### 4.2 Influence of soil constraint condition on the damage of vertical load-bearing components

#### 4.2.1 Component damage index

It can be concluded from the above that soil constraint conditions not only significantly influence the propagation characteristics of ground motion and the dynamic response of underground structures but also have a pronounced effect on the damage characteristics of critical vertical load-bearing components, such as columns.

To further analyze the influence of soil constraint conditions on the damage degree of such components, performance parameters should be selected to quantify the damage degree, which includes a variety of commonly adopted types, such as displacement, strain, and energy. Among these indexes, the section curvature, as a linking parameter between strain and component deformation, is more suitable for describing the component’s internal damage than displacement-type indexes and more convenient than strain-type or energy-type indexes in application [53]. Consequently, the curvature ductility coefficient *μ*_κ_ is adopted in this study as the index to quantify the damage degree of component sections, whose definition is as follows:

$$\mu_{\kappa }=\frac{\kappa_{p}}{\kappa_{y}}$$ (7)

where *κ*_y_ is the yield section curvature, and *κ*_p_ is the peak section curvature, as illustrated in Fig 13. In the case of *μ*_κ_<1, the section is not yield, while *μ*_κ_≥1 indicates that the section has yielded. The higher the *μ*_κ_, the higher the development degree of bending plastic hinges and the more severe the sectional damage.

#### 4.2.2 Component deformation and performance

**Fig 18:**
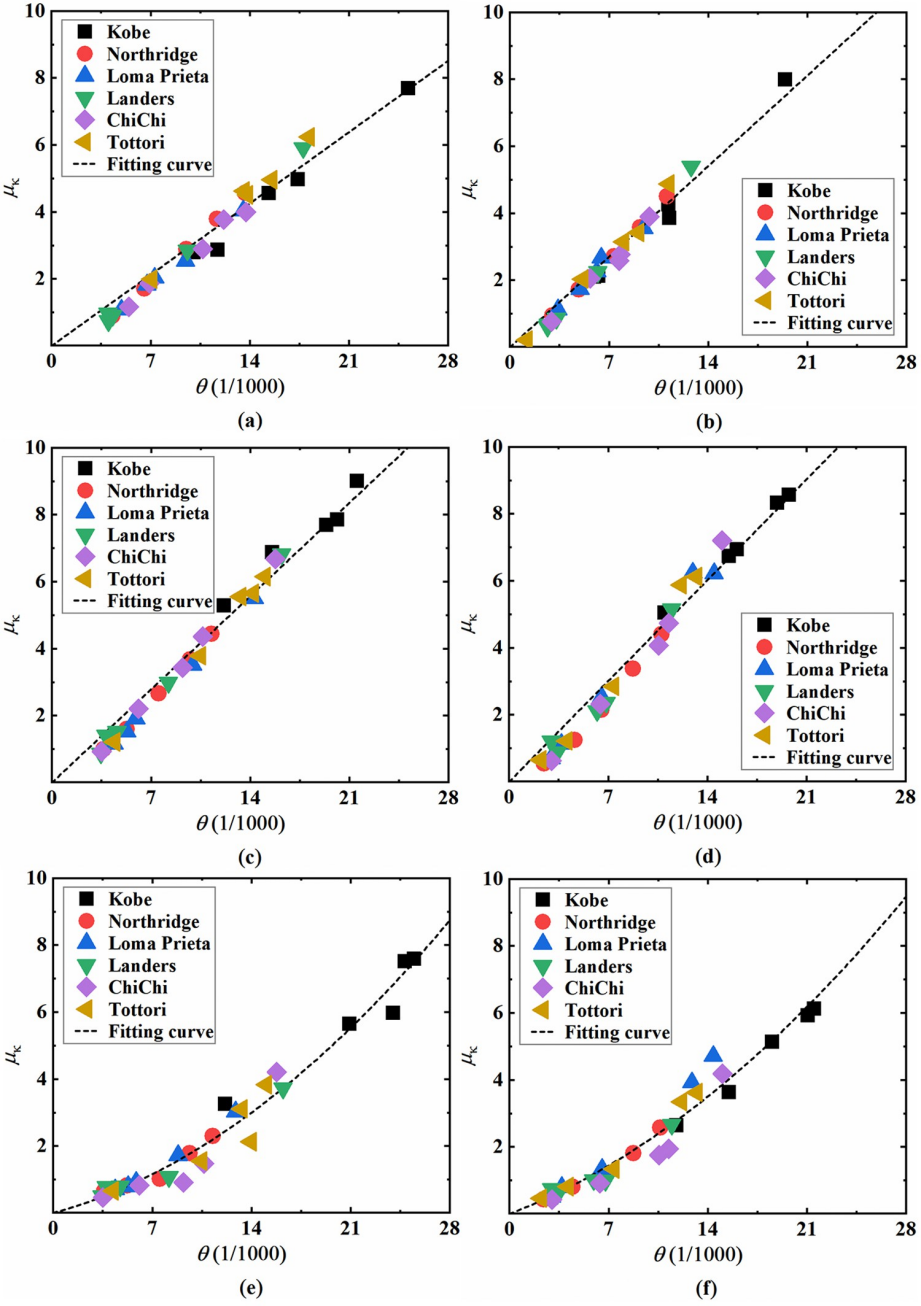
Bending deformation of component sections at site 3. (a) Top section of the double-storey station’s top column, (b) Top section of the three-storey station’s top column, (c) Bottom section of the double-storey station’s bottom column, (d) Bottom section of the three-storey station’s bottom column, (e) Bottom section of the double-storey station’s sidewall, (f) Bottom section of the three-storey station’s sidewall.

As mentioned above, among the vertical load-bearing components, the top section of top columns, the bottom section of bottom columns, and the bottom section of sidewalls are the weak positions of the underground station structures. Here, taking site 3 as an example, the curvature ductility coefficient *μ*_κ_ of the sections on these weak positions under different input seismic waves and the corresponding inter-storey drift ratio *θ* are plotted in Fig 18. Notably, the peak values before collapse are adopted for the analysis cases in which the structure collapses. The results of other sites are similar, which are not given due to space limitations.

As shown in Fig 18, the lateral deformation of station structures in most analysis cases exceeds the elastic-plastic inter-storey drift ratio limit for underground structures (1/250) by the standard for seismic design of underground structures (GB/T 51336–2018). For column sections, the bending deformation under different input seismic waves approximately increases linearly with the inter-storey drift ratio, and the results have a high correlation with the linear fitting line, as illustrated in Fig 18(A) to 18(D). The bending deformation at the bottom column’s bottom section is relatively higher than that of the top column’s top section, indicating that it is more susceptible to severe damage for the bottom columns in earthquakes. In addition, the column bending deformation of the three-storey station is higher than that of the double-storey station, especially at the top section of the top columns; that is, the increase in the total height and number of structure layers expands the damage risk of columns. For the bottom section of sidewalls, the bending deformation increases gently with increasing inter-storey drift ratio when the input seismic intensity is relatively low and then increases significantly along with increasing input intensity. The *μ*_κ_-*θ* curve of the sidewall section can be properly fitted with a quadratic polynomial, as shown in Fig 18(E) and 18(F). Moreover, the curves of the sidewall’s bottom section of the double-storey and three-storey station structures are found to be relatively similar, revealing that structural forms have little influence on the damage degree of sidewalls.

#### 4.2.3 Effect of soil constraint condition on component damage

**Fig 19:**
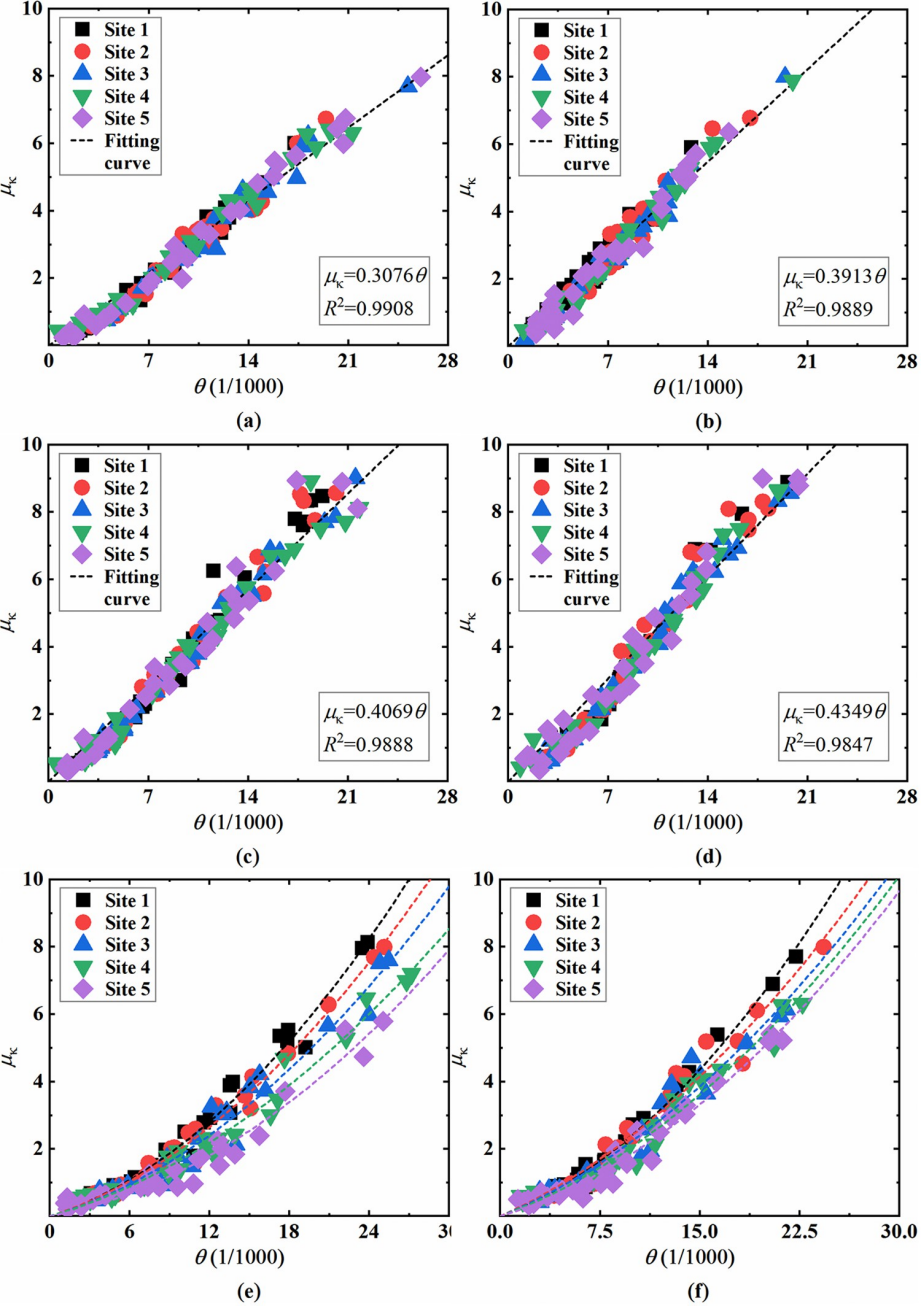
Component bending deformation at different sites. (a) Top column’s top section of the double-storey station, (b) Top column’s top section of the three-storey station, (c) Bottom column’s bottom section of the double-storey station, (d) Bottom column’s bottom section of the three-storey station, (e) Sidewall’s bottom section of the double-storey station, (f) Sidewall’s bottom section of the three-storey station.

The sectional bending deformation results under different soil conditions are further plotted in Fig 19. As shown, the curvature of column sections is generally linearly related to the inter-storey drift ratio under various analysis conditions. The linear fitting results of the bending deformation at the top column’s top section and the bottom column’s bottom section are given in Fig 19(A) to 19(D), whose corresponding determination coefficients (*R*^2^) of the fitting functions are all above 0.98, showing a high correlation. The results of different sites demonstrate no significant difference since the columns have no direct contact with the surrounding soil, and their deformation is mainly due to the seismic load transmitted by the beam and plate components. Therefore, soil constraint conditions have little influence on the damage of columns.

In Fig 19(E) and 19(F) and Table 5, the bending deformation at the sidewall’s bottom section and the corresponding fitting curves at different sites are given, from which it can be found that site conditions can significantly affect the deformation and damage of sidewalls. Specifically, the bending deformation decreases gradually along with the decrease of the soil-structure stiffness ratio. In softer site soil, the bending deformation at the sidewall’s bottom section is relatively minor, and the damage level is also lower under the same inter-storey drift deformation condition. Taking the double-storey station under the Northridge wave as an example, when the peak acceleration of the input seismic wave is 0.4 g, the inter-storey drift ratio of the station structure at site 1 reaches 1/207, and the corresponding ductility coefficient *μ*_κ_ of the sidewall’s bottom section is 0.913. At site 5, when the peak input acceleration is 0.6 g, the inter-storey drift ratio reaches 1/181, which is higher than the former analysis case. However, the *μ*_κ_ of the sidewall’s bottom section is only 0.754, which is significantly lower than that at site 1 with the relatively more solid site soil. When the inter-storey drift ratio reaches 1/250, the elastic-plastic drift ratio limit suggested by the standard for seismic design of underground structures (GB/T 51336–2018), the *μ*_κ_ at the sidewall’s bottom section are 0.399 and 0.589 for the double-storey and three-storey station at site 5. At the same time, those at site 1 are 0.644 and 0.747, which increased by 61.6% and 26.7%, respectively. The diversity reflects that the support and restraint of the surrounding soil have a significant impact on the sidewall’s deformation since the sidewalls are directly subjected to the dynamic action of the surrounding soil during earthquakes. Under the same inter-storey drift deformation level, when the soil-structure stiffness ratio decreases (i.e., the site soil is relatively softer), the damage degree of the sidewall’s bottom section falls. It is worth mentioning that this is incompatible with the study carried out by Yang et al. [11]. They suggested that when the soil-structure stiffness ratio is lower, the damage to vertical load-bearing components of underground structures is more serious. The discrepancy may be related to the fact that the concrete tensile damage factor was adopted as the component damage index in their study. The tensile damage of concrete material cannot fully reflect the functional characteristics of reinforced concrete structural components, considering that the vertical load-bearing components of underground structures are subjected to the combined action of axial and bending loads during earthquakes. It is suggested to comprehensively evaluate the damage and failure of structural components from the perspective of the global performance of component sections.

**Table 5:** Fitting functions of the bending deformation at the sidewall’s bottom section.

Structure type	Site type	*F*	Fitting function	*R* ^2^
Double-storey station	Site 1	10.26	*μ*_κ_ = 0.0091*θ*^2^+0.1247*θ*	0.9796
	Site 2	7.19	*μ*_κ_ = 0.0078*θ*^2^+0.1269*θ*	0.9832
	Site 3	4.66	*μ*_κ_ = 0.007*θ*^2^+0.1163*θ*	0.9637
	Site 4	2.68	*μ*_κ_ = 0.0057*θ*^2^+0.1134*θ*	0.9800
	Site 5	1.23	*μ*_κ_ = 0.0063*θ*^2^+0.0745*θ*	0.9579
Three-storey station	Site 1	9.48	*μ*_κ_ = 0.0094*θ*^2^+0.1492*θ*	0.9871
	Site 2	6.80	*μ*_κ_ = 0.0067*θ*^2^+0.1759*θ*	0.9681
	Site 3	4.56	*μ*_κ_ = 0.0063*θ*^2^+0.1621*θ*	0.9541
	Site 4	2.75	*μ*_κ_ = 0.0064*θ*^2^+0.1445*θ*	0.9693
	Site 5	1.37	*μ*_κ_ = 0.0067*θ*^2^+0.1206*θ*	0.9678

#### 4.2.4 Effect mechanism of soil constraint condition

**Fig 20:**
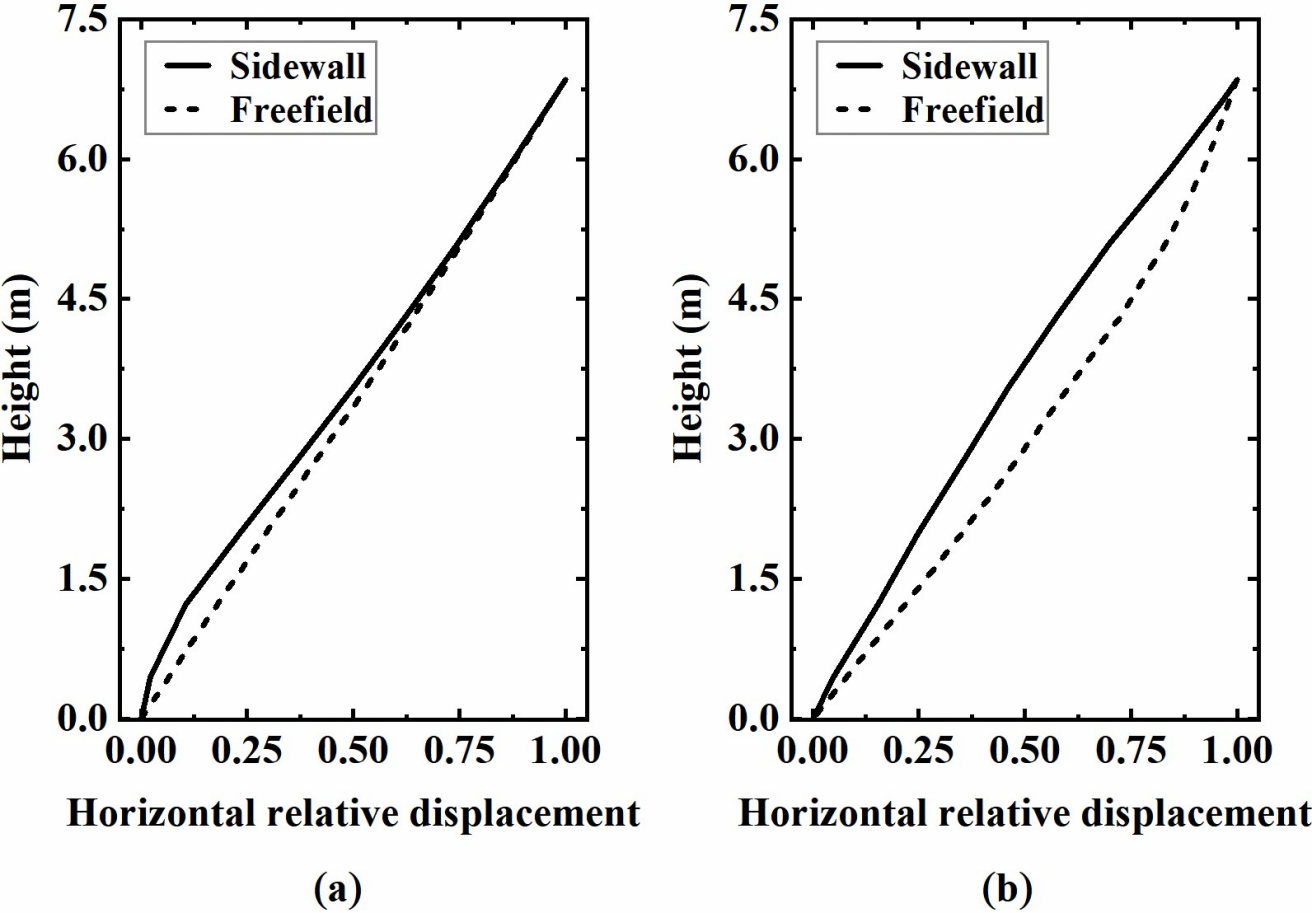
Horizontal displacement of the lower sidewall and free field soil. (a) Site 1, *F* = 10.26, (b) Site 5, *F* = 1.23.

To further explain the effect mechanism of the soil constraint on the sidewall deformation, the normalized horizontal displacement of the lower sidewall and the free field at the corresponding position in the analysis cases from the above section are visualized in Fig 20. As shown in Fig 20(A), the horizontal deformation form of the top half of the lower sidewall generally coincides with that of the free field at site 1, indicating that the soil with a relatively high soil-structure stiffness ratio has a substantial restraining effect on the underground structure. The sidewall deformation is "forced" to be consistent with that of the free field soil under seismic load, resulting in increased bending deformation and aggravated damage at the bottom of the sidewall. In contrast, at site 5, the horizontal deformation of the sidewall varies greatly from that of the free field soil, as shown in Fig 20(B). The soil constraint on the structure is much weaker due to the relatively low soil-structure stiffness ratio, which reduces the bending deformation and damage degree at the sidewall’s bottom. Meanwhile, this also illustrates apparent defects in adopting the inter-storey drift ratio alone as a measurement index of the aseismic performance for underground structures. The soil conditions and other factors should also be considered to achieve a comprehensive evaluation.

## 5 Conclusions

In order to investigate the deformation and failure characteristics of underground subway station structures under different soil constraint conditions in earthquakes, static-dynamic coupled nonlinear numerical analysis models of soil-structure interaction systems are established, and a series of dynamic analyses are conducted in this study. The damage process and failure mode of the double-storey and three-storey station structures are discussed according to the bending plastic hinge discriminant method based on the static analysis results of the vertical load-bearing components under different axial compression ratios. Furthermore, the influence of soil constraint conditions on the deformation and damage of vertical load-bearing components and the effect mechanism are quantitatively analyzed and discussed. The key findings of this study are summarized as follows:

1. Under the same inter-storey drift deformation level, noticeable differences are observed in the bending plastic hinge development and distribution of underground structures with different soil-structure stiffness ratios. It indicates that the deformation characteristics and internal force distribution of underground structures can be affected by the soil constraint conditions. Adopting the inter-storey drift ratio alone to quantify the damage degree of underground structures may result in erroneous seismic performance estimations.
2. The collapse of both the double-storey and three-storey station structures starts with the failure of the columns’ bottom sections, indicating that columns are the critical components in the progressive collapse of underground structures in earthquakes. Therefore, sufficient ductility or appropriate seismic isolation measures should be adopted in the seismic design of columns.
3. The bending deformation at the columns’ end sections is linearly related to the inter-storey drift ratio in general, and the soil constraint conditions have no apparent influence. The fitting curves between the section ductility ratio and inter-storey drift ratio based on the analysis results may provide a helpful reference for evaluating the ductility ratio requirements of columns in the seismic design of underground structures. To improve the prediction, more factors, such as soil stratification and component cross-section size, are suggested to be considered in future research.
4. With an increase in the soil-structure stiffness ratio, the bending deformation and damage degree at the sidewalls’ bottom sections increase at the same inter-storey drift deformation level since the deformation form of sidewalls is more consistent with that of the free field soil in a site with relatively higher soil-structure stiffness ratio. The sidewall bending ductility ratios of the double-storey and three-storey stations at the elastic-plastic drift ratio limit increased by 61.6% and 26.7%, respectively. It is suggested that the seismic design of sidewalls should be appropriately strengthened for the subway stations installed in sites with relatively high soil-structure stiffness ratios.

## Supporting information

S1 File Original data and plot for Fig 2 in the article. (OPJU)

S2 File Original data and plot for Fig 5 in the article. (OPJU)

S3 File Original data and plot for Fig 6A and 6B in the article. (OPJU)

S4 File Original data and plot for Fig 10 in the article. (OPJU)

S5 File Original data and plot for Fig 12 in the article. (OPJU)

S6 File Original data and plot for Fig 13 in the article. (OPJU)

S7 File Original data and plot for Fig 14 in the article. (OPJU)

S8 File Original data and plot for Fig 15 in the article. (OPJU)

S9 File Original data and plot for Fig 18A in the article. (OPJU)

S10 File Original data and plot for Fig 18B in the article. (OPJU)

S11 File Original data and plot for Fig 18C in the article. (OPJU)

S12 File Original data and plot for Fig 18D in the article. (OPJU)

S13 File Original data and plot for Fig 18E in the article. (OPJU)

S14 File Original data and plot for Fig 18F in the article. (OPJU)

S15 File Original data and plot for Fig 19A in the article. (OPJU)

S16 File Original data and plot for Fig 19B in the article. (OPJU)

S17 File Original data and plot for Fig 19C in the article. (OPJU)

S18 File Original data and plot for Fig 19D in the article. (OPJU)

S19 File Original data and plot for Fig 19E in the article. (OPJU)

S20 File Original data and plot for Fig 19F in the article. (OPJU)

S21 File Original data and plot for Fig 20 in the article. (OPJU)
